# Hearts from Mice Fed a Non-Obesogenic High-Fat Diet Exhibit Changes in Their Oxidative State, Calcium and Mitochondria in Parallel with Increased Susceptibility to Reperfusion Injury

**DOI:** 10.1371/journal.pone.0100579

**Published:** 2014-06-20

**Authors:** Ben Littlejohns, Philippe Pasdois, Simon Duggan, Andrew R. Bond, Kate Heesom, Christopher L. Jackson, Gianni D. Angelini, Andrew P. Halestrap, M.-Saadeh Suleiman

**Affiliations:** 1 Bristol Heart Institute, School of Clinical Sciences, Faculty of Medicine and Dentistry, University of Bristol, Bristol, United Kingdom; 2 Bioénergétique et Métabolisme, Institut de Rythmologie et Modélisation Cardiaque, Université de Bordeaux, Pessac, France; 3 Oxford Heart Centre, John Radcliffe Hospital, Oxford, United Kingdom; 4 Proteomics Facility, Faculty of Medical and Veterinary Sciences, University of Bristol, Bristol, United Kingdom; 5 School of Biochemistry, Faculty of Medical and Veterinary Sciences, University of Bristol, Bristol, United Kingdom; University of Pecs Medical School, Hungary

## Abstract

**Rationale:**

High-fat diet with obesity-associated co-morbidities triggers cardiac remodeling and renders the heart more vulnerable to ischemia/reperfusion injury. However, the effect of high-fat diet without obesity and associated co-morbidities is presently unknown.

**Objectives:**

To characterize a non-obese mouse model of high-fat diet, assess the vulnerability of hearts to reperfusion injury and to investigate cardiac cellular remodeling in relation to the mechanism(s) underlying reperfusion injury.

**Methods and Results:**

Feeding C57BL/6J male mice high-fat diet for 20 weeks did not induce obesity, diabetes, cardiac hypertrophy, cardiac dysfunction, atherosclerosis or cardiac apoptosis. However, isolated perfused hearts from mice fed high-fat diet were more vulnerable to reperfusion injury than those from mice fed normal diet. In isolated cardiomyocytes, high-fat diet was associated with higher diastolic intracellular Ca^2+^ concentration and greater damage to isolated cardiomyocytes following simulated ischemia/reperfusion. High-fat diet was also associated with changes in mitochondrial morphology and expression of some related proteins but not mitochondrial respiration or reactive oxygen species turnover rates. Proteomics, western blot and high-performance liquid chromatography techniques revealed that high-fat diet led to less cardiac oxidative stress, higher catalase expression and significant changes in expression of putative components of the mitochondrial permeability transition pore (mPTP). Inhibition of the mPTP conferred relatively more cardio-protection in the high-fat fed mice compared to normal diet.

**Conclusions:**

This study shows for the first time that high-fat diet, independent of obesity-induced co-morbidities, triggers changes in cardiac oxidative state, calcium handling and mitochondria which are likely to be responsible for increased vulnerability to cardiac insults.

## Introduction

High-fat diet causes cardiac alterations which can be the result of direct effects on the heart (e.g. by altering cardiac metabolism) or indirectly as a result of obesity and associated pathologies (e.g. diabetes, hypertension, cardiac hypertrophy, ischemia, fibrosis and heart failure) [1]–[3]. Obesity triggers triglyceride accumulation and the formation of pro-apoptotic ceramides within cardiomyocytes which have been implicated in the impairment of contractile dysfunction [4]–[7] and possibly causing insulin resistance [4].

Whether obesity induces changes in cardiac function varies depending on the diet composition, duration of feeding and the experimental model. Uncoupling of oxidative phosphorylation and the production of reactive oxygen species (ROS) could combine to induce contractile dysfunction [8] (reviewed in [3]) possibly by alterations in Ca^2+^ cycling [9]–[11] and/or to lower ATP/oxygen ratio associated with fatty acid oxidation [12]. In addition to changes in Ca^2+^ cycling, lipid accumulation, apoptosis, oxidative stress and mitochondrial dysfunction are also potential contributors to contractile dysfunction [13]. Whether these changes are a result of obesity and the related co-morbidities or directly due to the high-fat diet has not been established. High-fat diet triggers cardiac mitochondrial abnormalities (functional and structural) under normal and pathological conditions [14], [15] which includes an increase in mitochondrial permeability transition pore (mPTP) opening in interfibrillar mitochondria [16], [17]. The latter could explain why feeding rodents an obesity-generating high-fat diet increases vulnerability of hearts to ischemia/reperfusion (I/R) [18]–[20] whilst lipid-lowering drugs reduce the incidence of ischemia-induced ventricular arrhythmias and decrease infarct size after I/R [21], [22]. Significant Ca^2+^ overload and oxidative stress are triggers of reperfusion injury that lead to mPTP opening [23], [24]. Overall, it is evident that obesity-induced chronic and complex metabolic, functional and structural changes in the heart will render the myocardium more vulnerable to I/R injury. The vulnerability is independent of whether the perfusate contains lipids or not [25].

Experimental models used to investigate cardiac remodeling associated with high-fat diet are either genetically altered or employ wild-type animals fed high-fat diet. Genetic models provide important information but they have their limitations [26]. In the majority of wild-type animal models, rodents are fed high-fat diet with fat caloric content ranging from 20% to 60% [8], [12], [18], [26]–[29]. A standard chow diet would normally contain fat providing about 12% of total calories (∼5% by weight). Depending on its composition, high-fat diet can result in excessive, moderate or little weight gain after weeks (or months) of feeding (e.g. [8], [12], [15], [26], [27], [29], [30]). For example high-fat diet containing high sucrose carbohydrates would induce excessive weight gain, diabetes and cardiac dysfunction (e.g. [30]–[32]). Sucrose is critical in triggering obesity and/or diabetic phenotype in rats in the presence [33] or absence of high-fat diet [34]. Additionally, dietary sucrose (compared to starch) is associated with obesity, insulin insensitivity, hyperinsulinemia and higher serum lipid and glucose levels [35], [36]. Although excessive weight gain can cause hypertension, the effect is very small in rodents where even obese mice and rats have little or mild increase in blood pressure [27], [28].

The C57BL/6 mouse strain is used as a model for studies of diet-induced atherosclerosis and/or obesity and diabetes. These mice become obese, hyperglycemic and insulin resistant when fed certain types of high-fat diet (e.g. [27], [32]) but do not gain extra weight or show diabetic phenotype when fed high-fat atherogenic diet (e.g. containing cholesterol and low sucrose) and become susceptible to atherosclerotic lesion development after long periods of feeding [30], [37]. The overall aims of this research were to characterize a non-obese mouse model fed high-fat diet and to determine whether the associated cardiac remodeling of mechanisms underlying I/R injury (oxidative stress, Ca^2+^ handling and mPTP) can explain altered vulnerability of hearts and cardiomyocytes to cardiac insults.

## Methods

### Animals and diet

Breeding, maintenance and feeding of C57BL/6J male mice as well as weight monitoring and clinical chemistry were all carried out at Charles River facilities (Charles River, Margate, Kent, UK). The diet (see below) was delivered directly from the supplier to Charles River. At the end of the feeding protocols, mice were delivered and housed for a minimum period of one week at the Animal Services Unit, University of Bristol. C57BL/6J male mice were allocated to one of two feeding protocols. During the feeding protocols the mice were given *ad libitum* access to food and water and maintained on a 12 h light/dark cycle. Mice were fed standard murine chow diet post weaning until 6 weeks of age. The standard murine chow diet contained 13% calories from fat, 22% calories from protein and 65% calories from carbohydrate (Special Diets Services, UK, code: 801900; http://www.sdsdiets.com/pdfs/VRF1-P.pdf). At 6 weeks of age they were either continued on standard diet (normal diet mice) or switched to high-fat diet (high-fat diet mice) for a further 20–21 weeks. The high-fat diet consisted of 45% calories from fat, 18% calories from protein and 37% calories from carbohydrate (Special Diets Services, UK, code: 821424). The high-fat diet also contained 0.17% calories from cholesterol and low sucrose content. The dietary fat was from lard and consisted of a mixture of saturated (44%) and mono- (43%) and poly-unsaturated (13%) fatty acids. More details of high-fat diet composition are shown in Table S1. The gross energy for high-fat and normal diets was 19.67 and 16.54 kJ·g^−1^, respectively. Crude fat content (by weight) in different batches ranged between 21–23%. This high-fat diet is known to promote atherosclerosis in transgenic mice models without inducing significant body weight gain [38]–[41].

### Ethics Statement

Animal work was performed in accordance with the UK Animals (Scientific Procedures) Act of 1986 and approved by the University of Bristol Animal Welfare and Ethical Review Board (Permit numbers: PPL 30/2859 and PIL 30/6547).

### *In vivo* measurements

#### Clinical chemistry measurements

Tail vein blood was taken from non-fasted mice and pooled (one pooled sample was from three animals) to measure cholesterol, triglycerides and glucose performed by Charles River (Margate, UK). An intra-peritoneal insulin tolerance test (IPITT) was also performed on animals from the two groups (approx. 25 weeks old). For this purpose, blood glucose was measured from the tail vein of mice that had been fasted for 4 h. Insulin was then administered by an intra-peritoneal injection at a concentration of 1 IU·kg^−1^ and blood glucose was measured at 15, 30, 60 and 120 min post insulin injection.

#### Echocardiography

2-D echocardiography was performed using a Vevo 770 High-Resolution *In Vivo* Imaging System (VisualSonics, Canada) on mice one week before use for other experiments (approx. 25 weeks old). Mice were anesthetized by inhalation of isoflurane and kept warm using a heat pad set at 37°C. M-mode recordings of the hearts were taken using a parasternal short axis view at the level of the papillary muscles. For each heart at least five separate M-mode measurements were taken. Following anesthesia mice were closely monitored until full recovery was observed. Heart rates for the majority of anesthetized mice were in the range of 400–475 bpm. Mice with heart rates outside this range were excluded from the analysis.

#### Histology

Following a ventral midline thoracotomy, and incision of the jugular veins, mice were perfused and fixed at physiological pressure, via the left ventricle, with 10% formalin. Tissue was excised and post-fixed in formalin for at least 16 h prior to histological processing [42]. Sections were stained with elastic van Gieson for morphology and identification of lesions in aortic sinuses, coronary arteries and brachiocephalic arteries from mice fed high-fat diet.

### Experiments on isolated hearts

#### Langendorff perfusion

Hearts were excised from freshly sacrificed mice, placed in ice cold Krebs-Henseleit buffer, cannulated via the aorta onto a Langendorff system (ADInstruments, UK) and perfused with 37°C Krebs-Henseleit buffer, consisting of, in mM, 120 NaCl, 25 NaHCO_3_, 11 D-glucose anhydrous, 1.2 KH_2_PO_4_, 1.2 MgSO_4_·7H_2_O, 4.8 KCl and 2 CaCl_2_. The buffer solution in the reservoirs was gassed with 95% O_2_ and 5% CO_2_ (pH 7.4). Hearts were perfused with a constant filling pressure of 60 mmHg and coronary flow monitored throughout.

#### Ischemia/reperfusion protocol

The hearts were perfused with Krebs-Henseleit buffer for a 30 min stabilization period, followed by 40 min of no flow global ischemia and 120 min reperfusion. For experiments using cyclosporin A (CsA) the drug was present 10 min before ischemia and remained in the perfusate until 20 min into reperfusion. The CsA was dissolved in DMSO (1∶10,000 final dilution) and added to the buffer at a concentration of 0.2 µM. During the experiment hearts were bathed in 37°C buffer and coronary flow rate and filling pressure were measured by Chart 5 software (ADInstruments, UK).

#### Myocardial injury

Triphenyl tetrazolium chloride (TTC) staining was used to determine the infarct size within the heart as described previously [43]. Briefly, at the end of reperfusion hearts were perfused with a 1% (w/v) TTC PBS solution for 10 min, frozen and then cut into 5 equally sized transverse slices. Infarct size was calculated using ImagePro Plus software (Media Cybernetics, USA). In addition to cardiac injury, vascular dysfunction was also monitored by comparing the extent of recovery in coronary flow rate following I/R.

#### Western blotting

Ventricular tissue was collected from freshly sacrificed mice or following I/R, snap frozen in liquid nitrogen and stored at −80°C. Tissue was homogenized in 10 µL radio-immunoprecipitation assay (RIPA) buffer per mg wet weight and centrifuged at 10,000×g for 10 min at 4°C. Proteins in the supernatant or mitochondrial proteins (10 µg) were separated using SDS-polyacrylamide gel electrophoresis under reducing and denaturing conditions and transferred to a 0.45 µm polyvinylidene difluoride membrane. The membranes were blocked with tris-buffered saline (TBS)-Tween, containing either 10% (w/v) skimmed milk powder or 5% (w/v) BSA, before incubation overnight (4°C) with a primary antibody diluted in TBS-Tween containing 5% (w/v) BSA. Primary antibodies used included phosphorylated (Ser473) Akt (1∶2000, Cell Signaling), Akt (1∶2000, Cell Signaling), cleaved caspase 3 (CC3) (1∶1000, Cell Signaling), Bcl-2-associated X protein (BAX) (1∶2000, Cell Signaling), B-cell lymphoma-2 (Bcl-2) (1∶2000, Cell Signaling), mitofusin 1 (Mfn-1) (1∶1000, Abcam), Mfn-2 (1∶1000, Abcam), optic atrophy 1 (OPA1) (1∶10,000, Abcam), dynamin related protein 1 (DRP1) (1∶1000, Cell Signaling), phosphate carrier (PiC) (1∶100,000, Sigma- Genosys), voltage-dependent anion channel (VDAC) (1∶4000, Cell Signaling), cyclophilin D (CypD) (1∶2000, Abcam), adenine nucleotide transferase (ANT) (1∶10,000, custom made (see [44])), hexokinase II (1∶1000, Cell Signaling), phosphorylated (Ser16) phospholamban (P-PLN) (1∶2000, Abcam), PLN (1∶5000, Abcam) and catalase (1∶2000, Abcam). For whole heart tissue glyceraldehyde-3-phosphate dehydrogenase (GAPDH) was used as a loading control (1∶10,000, Cell Signaling) and for mitochondrial fractions total protein blots were used. Blots were then incubated with an appropriate horseradish peroxidase conjugated secondary antibody (1∶10,000, GE Healthcare Life Sciences) and proteins were visualized using the enhanced chemiluminescence system. Protein bands were quantified by densitometry with ImageJ 1.46r software.

#### Malondialdehyde assay

Cardiac malondialdehyde (MDA) was measured using high-performance liquid chromatography (HPLC) on a 4 µm Nova-Pak C18 column (150 mm×3.9 mm) (Waters, UK) as described elsewhere [45]. In brief, the extract was prepared by adding 50 µL of 6 M NaOH to 250 µL of 0.8 mg·mL^−1^ tissue sample diluted in RIPA buffer and incubated at 60°C for 30 min. The protein was then precipitated with 125 µL of 35% (v/v) perchloric acid and the mixture was centrifuged at 2800×g for 10 min. 250 µL supernatant of each standard and sample was mixed with 25 µL 2,4-dinitrophenylhydrazine (prepared as a 5 mM solution in 2 M HCl). This mixture was incubated for 30 min at room temperature in the dark and then centrifuged at 4000×g for 5 min. 50 µL of standards and samples were injected into the HPLC system. The mobile phase was 12.4% (v/v) acetic acid and 38% (v/v) acetonitrile and was perfused through the column at a flow rate of 0.6 mL·min^−1^ at room temperature. Chromatograms were acquired by measuring absorbance at 310 nm.

#### Proteomics

Protein analysis was performed by the proteomics facility, School of Medical Sciences, University of Bristol. Isobaric Tandem Mass Tags (TMTs) (ThermoFisher Scientific, UK) with an amine-reactive moiety were used for analysis of protein expression in extracted cardiac tissue. Each sample was initially digested with trypsin (0.025 µg·µg of protein^−1^ at 37°C overnight, Promega, UK) and then labelled with TMT sixplex reagents according to the manufacturer's protocol (Thermo Scientific UK). The samples were then analyzed by reverse phase nano-liquid chromatography mass spectrometry/mass spectrometry using a LTQ-Orbitrap Velos mass spectrometer (Thermo Scientific, UK). The peptide fragments released the isobaric tags which were used to provide quantification of the peptides. The peptides were searched against the UniProt/SwissProt mouse database (81,998 entries) using the SEQUEST (Ver. 28 Rev. 13) algorithm to determine the source protein. Protein quantitation was the median value of peptide(s) identified from the same protein. The peptides were analyzed using Thermo Proteome Discoverer 1.2.0.208 software (ThermoFisher Scientific, UK) and quantified proteins were recorded as a ratio to create fold change against a standard sample (created by pooling an equal volume from each sample) and normalized to GAPDH.

#### Electron microscopy

Hearts were excised from freshly sacrificed mice, cannulated via the aorta and retrogradely perfused with 37°C Krebs-Henseleit buffer, as in the Langendorff perfusion section. The hearts were perfused for 5 min with buffer and then for 2 min with the fixative solution at a rate of 1 mL·min^−1^. The fixative solution was made up of 0.1 M phosphate buffer (22.5 mM NaH_2_PO_4_·2H_2_O and 76.76 mM Na_2_HPO_4_, pH 7.4) with the following added to it: 0.5 mM CaCl_2_, 1.7 mM D-glucose anhydrous, 1% (v/v) glutaraldehyde from a 25% stock solution and 1% (w/v) paraformaldehyde. Subsequently, a ∼2 mm slice was taken after removing the apex of the heart. The heart slices were stored overnight at 4°C in fixative solution. This was followed by more washing and dehydration and the final part of the fixation was to embed the tissue in EPON epoxy resin and polymerize at 60°C for 48–72 h. Thick sections (2.5 µm) were cut with a microtome (Leica, Germany) so that the cardiomyocytes were orientated into a longitudinal plane. Thin sections (70 nm) were cut and stained with uranyl acetate and lead citrate (94 mM lead nitrate, 140 mM sodium citrate and 0.19 mM NaOH) and viewed with a Tecnai 12 bioTWIN transmission electron microscope (FEI, Netherlands) in the Wolfson Bioimaging Facility at the University of Bristol. Images were taken with an Eagle 4K×4K charged coupled device camera (FEI, Netherlands). From the electron micrographs the area, lengths and density (mitochondrial coverage of total myofilament area) of interfibrillar mitochondria were measured using ImagePro Plus Version 6.2.0.424 software (Media Cybernetics, USA).

### Experiments on isolated cardiomyocytes

#### Cardiomyocyte isolation

Ventricular cardiomyocytes were isolated using collagenase (1 g·L^−1^, type-1, Worthington Biochemical Corporation, New Jersey, USA) and protease (0.05 g·L^−1^, type XIV, Sigma-Aldrich, Dorset, UK) digestion as described previously [46]. The only modification was that the perfusion speed was 2.3 mL·min^−1^ and there was no CaCl_2_ in the enzyme solution. Once digested, the ventricular tissue was separated and mechanically dispersed by shaking at 37°C. Cardiomyocytes were filtered and then the CaCl_2_ concentration in the buffer was gradually increased to a final concentration of 1 mM.

#### Superfusion and stimulation of cardiomyocytes

Cardiomyocytes were superfused at a rate of 1.3 mL·min^−1^ with HEPES buffer solution consisting of, in mM, 137 NaCl, 5 KCl, 1.2 MgSO_4_·7H_2_O, 1.2 NaH_2_PO_4_·2H_2_O, 20 HEPES, 15 D-glucose anhydrous and 2 CaCl_2_ (pH 7.4) [47]. The microscope stage was heated by a temperature controller so that the solution bathing the cells was 32–33°C. The stimulation voltage was set at just above the threshold required for the cell to beat.

#### Changes in cardiomyocyte morphology during metabolic inhibition

Cardiomyocytes were initially superfused with HEPES buffer solution and field stimulated at 0.2 Hz throughout the protocol. The solution superfusing the cells was switched from HEPES buffer to HEPES buffer containing 2 mM NaCN and no glucose (metabolic inhibition) which has been shown to mimic the effects of hypoxia [47], [48]. After a period of metabolic inhibition the cardiomyocyte goes into rigor. After 10 min in rigor, the perfusion was switched back to HEPES buffer (reperfusion) for 10 min. Parameters such as time to stop beating, time to rigor and contractile recovery after reperfusion were recorded.

#### Intracellular Ca^2+^ measurements

Isolated cardiomyocytes were loaded with the fluorescent dye Fura-2 AM ester (Biotium Inc., USA) at a concentration of 2 µM and gently shaken at 37°C for 15 min. Loaded cardiomyocytes were superfused with HEPES buffer, stimulated at different frequencies and the intracellular Ca^2+^ concentrations ([Ca^2+^]_i_) were measured (for a representative trace see Results section). The ratio of 340∶380 was used as an indication of [Ca^2+^]_i_. The excitation wavelengths were set so that 20 ratio measurements were taken per second. The excitation dichroic mirror was 415 nm and the emission passed through a 510±20 nm bandpass filter. The photomultiplier was connected to Felix 32 Analysis version 1.2 software (Photon Technology International, USA).

#### ROS turnover in a cardiomyocyte suspension

The fluorescent dye 5-(and 6)-chloromethyl-2′, 7′-dichlorodihydrofluorescein diacetate (CM-H_2_DCFDA) (5 µM) (Life Technologies, UK) was added to a cardiomyocyte suspension for 15 min at room temperature. After loading, the solution was centrifuged at 100×g for 1 min at room temperature and the cell pellet was resuspended in HEPES buffer or HEPES buffer without glucose but supplemented with 0.5 mM palmitate bound to 1% (w/v) fatty acid free BSA, as described previously [49]. 200 µL aliquots of the cardiomyocyte suspension were added to a 96-well plate, excited at 485 nm and fluorescence was detected at 520 nm using a fluorescent plate reader (37°C) (FLUOstar Optima, BMG Labtech, Germany).

### Experiments on isolated mitochondria

#### Mitochondrial isolation

Two methods were used to isolate mitochondria, a protease method and a Polytron method, as described previously for rat heart mitochondria [50]. The protease method was preferred as it yielded more mitochondria; however, if outer mitochondrial membrane proteins were to be analyzed, proteolytic cleavage of the proteins might occur and so the Polytron method was used. Hearts were excised from freshly sacrificed mice, cannulated via the aorta and perfused with isolation buffer to wash out blood. The isolation buffer consisted of 300 mM sucrose, 10 mM Tris-HCl and 2 mM EGTA and the pH was 7.2 at 4°C. For the Polytron method the isolation buffer was supplemented with protease (cOmplete EDTA-free, Roche, UK) and phosphatase (phosphatase inhibitor cocktail 3, Sigma, UK) inhibitors. The centrifuge steps were carried out as in [50] with density-gradient purification of mitochondria in 25% (v/v) Percoll.

#### Oxygen consumption rates in isolated cardiac mitochondria

Mitochondria prepared with the protease method were used to determine the O_2_ consumption rates at 37°C using a High-Resolution Respirometry Oxygraph-2K (Oroboros Instruments, Austria), as described previously [51]. Mitochondria (0.125 mg·mL^−1^ final) were added to 2 mL of KCl buffer in the chamber and the O_2_ consumption was recorded. The KCl buffer contained, in mM, 125 KCl, 20 MOPS, 10 Tris, 0.01 EGTA, 2.5 KH_2_PO_4_, 2.5 MgCl_2_ and 2% (w/v) fatty acid free BSA. The buffer was set to pH 7.1 at 37°C with KOH. The O_2_ consumption was recorded in state 2 to assess basal respiration, state 3.5 thought to mimic ATP turnover *in vivo* and state 3 respiration to assess maximal respiration rates. To obtain state 2 respiration mitochondria (0.125 mg·mL^−1^) were added to the chamber containing the KCl buffer. For respiration linked to NADH oxidation 5 mM pyruvate and 2.5 mM L-malate were added and for respiration linked to fatty acid β-oxidation 10 µM palmitoylcarnitine was added instead of pyruvate and the L-malate concentration was reduced to 1 mM. When a steady state of O_2_ consumption was reached a measurement of state 2 was taken. From the stable rate the O_2_ consumption was determined in either state 3.5 or state 3. For state 3.5 respiration 5 mM creatine, 40 µg creatine kinase and 400 µM ATP were added to the chamber. To obtain state 3 respiration 1.5 mM ADP was added. When the O_2_ consumption again reached a stable rate 10 µM (final concentration) cytochrome c was added to determine the amount of outer mitochondrial membrane damage.

#### Hydrogen peroxide production by isolated cardiac mitochondria

Mitochondria prepared using the protease method were used to measure the rate of hydrogen peroxide production in state 3.5 from respiration linked to NADH oxidation and fatty acid β-oxidation (as above) and supplemented with 30 µM Amplex Red and 0.1 mg·mL^−1^ peroxidase, as previously described [51]. The samples were excited at 540 nm and emission measured at 585 nm with a multi-plate fluorescent plate reader at 37°C (Flexstation plate reader, Molecular Devices, USA).

#### Hexokinase activity assay

Hexokinase assay was performed on isolated mitochondria prepared using the Polytron method, as described previously [50]. The lysis buffer contained 33 mM KH_2_PO_4_, 50 µM dithiothreitol, protease inhibitor (cOmplete, mini, EDTA-free protease inhibitor cocktail, Roche, UK) and pH 7.2. The assay was performed at 37°C and the mitochondria were diluted to 2 mg·mL^−1^. The hexokinase buffer consisted of 100 mM Tris-HCl (pH 7.4) containing 0.4 mM NADP^+^, 10 mM MgCl_2_, 5 mM ATP and 0.3% (w/v) Triton X-100. Mitochondria (40 µg or 80 µg) were added into a cuvette containing 1 mL final volume of hexokinase buffer supplemented with 0.5 units·mL^−1^ glucose-6-phosphate dehydrogenase. The reaction was started after 2 min by addition of 1 mM glucose. For one mole of glucose used by hexokinase there was one mole of NADPH produced and therefore absorbance was recorded at 340 nm for 2 min with a spectrophotometer (Thermo Scientific, UK).

#### Citrate synthase activity assay

Citrate synthase activity assay was performed on isolated mitochondria prepared using the Polytron method, as described previously [50]. The assay was performed at 37°C and the mitochondria were diluted to 0.1 mg·mL^-1^ in lysis buffer, as above. The citrate synthase buffer consisted of 50 mM Tris-HCl (pH 7.4), 150 µM DTNB, 0.3% (w/v) Triton X-100 and pH 7.4. Mitochondria (2 µg or 4 µg) were added to a cuvette containing 1 mL final volume of citrate synthase buffer supplemented with 0.3 mM acetyl CoA. The samples were incubated at 37°C for 2 min, a blank measurement taken and then 0.5 mM oxaloacetate was added. Absorbance was recorded at 412 nm for 2 min with a spectrophotometer (Thermo Scientific, UK).

### Data analysis

Data were analyzed using Prism 5 Version 5.01 software (GraphPad, USA) and presented as mean±SEM where appropriate. Data were tested for normal distribution using the Kolmogorov-Smirnov test and equal variance using the F-test. Student's t-test was performed on data that were normally distributed and had equal variance. Student's t-test with Welch's correction was performed on data that were normally distributed but had unequal variance. The Mann-Whitney test was performed on data that were not normally distributed. Fisher's exact test was used for categorical data. Two-way ANOVA with the Bonferroni post-hoc test was used on data with two independent variables. Statistical tests were performed as paired or unpaired where appropriate. All statistical tests were performed as two-tailed and a P-value less than 0.05 was assumed to be significantly different.

## Results

Feeding C57BL/6J mice high-fat diet for 20 weeks resulted in elevated blood cholesterol and triglycerides and was associated with a small (∼3%) but significant increase in body weight compared to mice fed normal diet (Table 1). Most of the body weight gain was accounted for in the increased epididymal fat pad weights (Table 1).

**Table 1:** Characteristics of mice fed normal or high-fat diet.

Measurement	Normal Diet	High-Fat Diet
Body Weight (g)	31.2±0.2 (n = 158)	32.2±0.3 (n = 153)
Epididymal Fat Pad Weight (g)	0.53±0.02 (n = 55)	1.19±0.11*** (n = 34)
Blood Cholesterol (mM)	3.25±0.20 (n = 4)	5.06±0.52* (n = 4)
Blood Triglycerides (mg·dL^−1^)	120±15 (n = 4)	205±26* (n = 4)
Non-Fasting blood Glucose (mM)	9.26±0.64 (n = 4)	7.54±0.72 (n = 4)
Area Under the IPITT Curve (mM·min)	1332±149 (n = 6)	1349±186 (n = 6)
Wet Heart Weight/Body Weight (%)	0.70±0.13 (n = 55)	0.68±0.20 (n = 34)
Dry Heart Weight/Body Weight (%)	0.108±0.06 (n = 17)	0.108±0.012 (n = 20)

IPITT = intra-peritoneal insulin tolerance test. Data are presented as mean±SEM. *** = P<0.001 and * = P<0.05 vs. normal diet. Numbers shown in parenthesis indicate number of mice used except for cholesterol, triglycerides and glucose where n refers to number of measurements each containing a pool of 3 samples from 3 separate mice.

### The effect of high-fat diet on insulin resistance, atherosclerosis, cardiac pump function, cardiac hypertrophy and cardiac apoptosis

There was no evidence for a diabetic phenotype in the high-fat diet group as shown by similar non-fasting blood glucose and confirmed using an intra-peritoneal insulin tolerance test (Table 1). Histological studies demonstrated that despite elevated blood lipids the aortic sinus, brachiocephalic artery and coronary arteries had no lesions even after longer periods of high-fat feeding (Figure S1 A–C). There were no signs of cardiac hypertrophy in the high-fat diet group compared to the normal diet group as shown by wet and dry heart weight to body weight ratios (Table 1). Echocardiographic measurements showed that cardiac pump function (ejection fraction and fractional shortening) were similar for both groups and no difference in the left ventricular mass (Figure S1 D–E and Table 2).

**Table 2:** Echocardiography measurements taken from mice anesthetized with isoflurane.

Measurement	Normal Diet (n = 7)	High-Fat Diet (n = 7)
Left Ventricular Mass (mg)	152±7	132±7
Ejection Fraction (%)	75±1	74±2
Fractional Shortening (%)	43±1	42±2

Data was obtained from M-mode echocardiographic images taken in parasternal short axis mode at the level of the papillary muscles. Data are presented as mean±SEM. There was no statistical significance between the data.

There was no evidence for increased apoptosis in the high-fat diet group as measured by pro- and anti-apoptotic protein levels including Akt phosphorylation, cleaved-caspase 3, BAX and Bcl-2. This was also confirmed by measuring BAX/Bcl-2 ratio and mitochondrial BAX (Figure S2).

Overall, apart from high blood triglycerides and cholesterol levels and increased epididymal fat deposits, mice fed high-fat diet did not have obesity associated co-morbidities and had normal cardiac pump function. As this diet does not induce marked body weight and was not associated with any of the known obesity-induced co-morbidities, we shall refer to it as non-obesogenic high-fat diet.

### The effect of non-obesogenic high-fat diet on vulnerability of hearts to I/R

**Figure 1:**
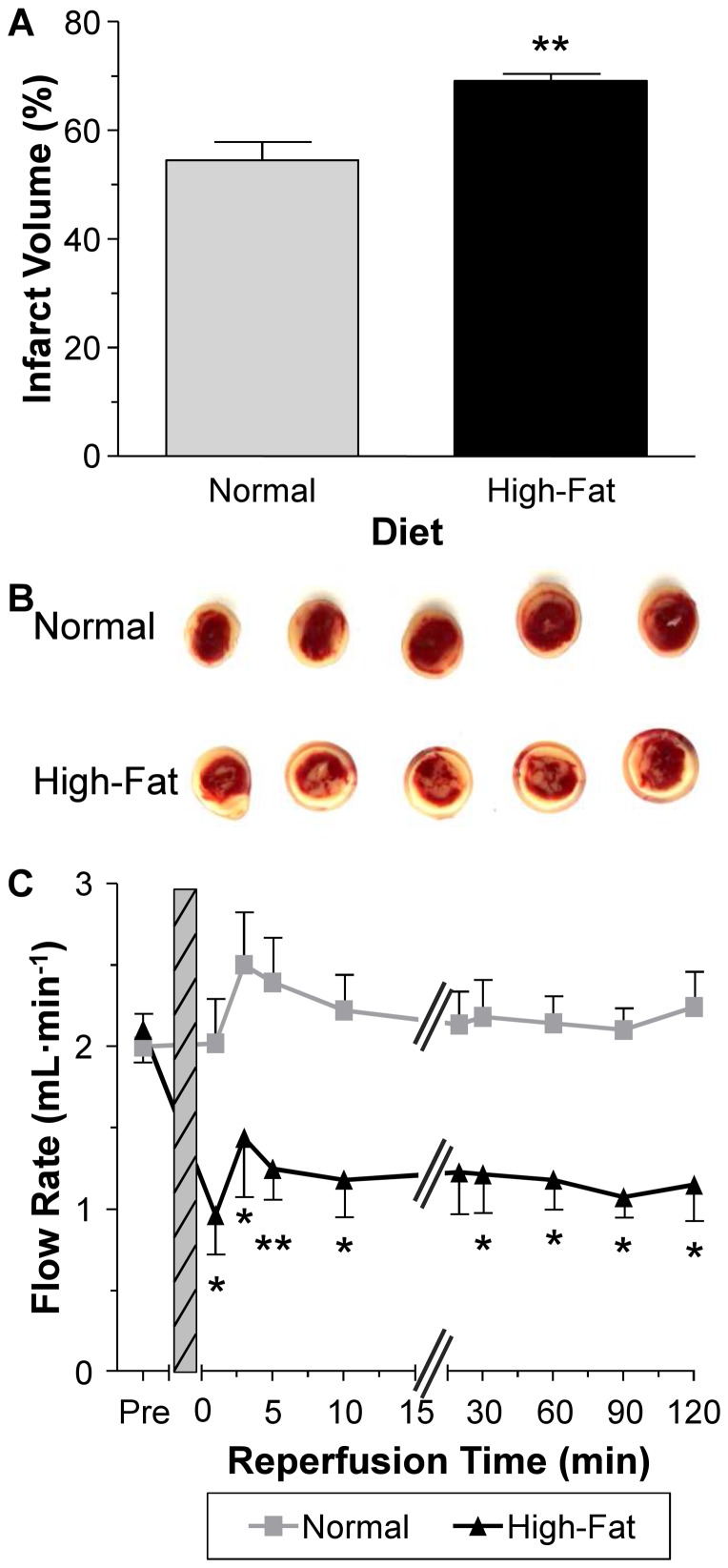
Markers of injury during I/R. A) Infarct volume quantified from the heart slices stained with TTC. B) Representative heart slices from the normal diet group and high-fat diet group. C) Flow rate determined at different time points during the reperfusion phase. Pre = pre-ischemia and grey hashed area = 40 min ischemia. Data are presented as mean±SEM (n = 6-7 hearts). Data were analyzed using Student's t-test (A) and two-way repeated measures ANOVA with the Bonferroni post-hoc test (C). ** = P<0.01 and * = P<0.05 vs. normal diet.

Hearts subjected to I/R had significantly more infarct volume in the high-fat diet group compared to those in the normal diet group, P<0.01 (Figure 1 A–B). The pre-ischemic flow rate values were similar for both groups but significantly lower at the end of reperfusion in the high-fat diet group compared to the normal diet group, P<0.05 (Figure 1 C).

### The effect of non-obesogenic high-fat diet on vulnerability of cardiomyocytes to metabolic inhibition

**Figure 2:**
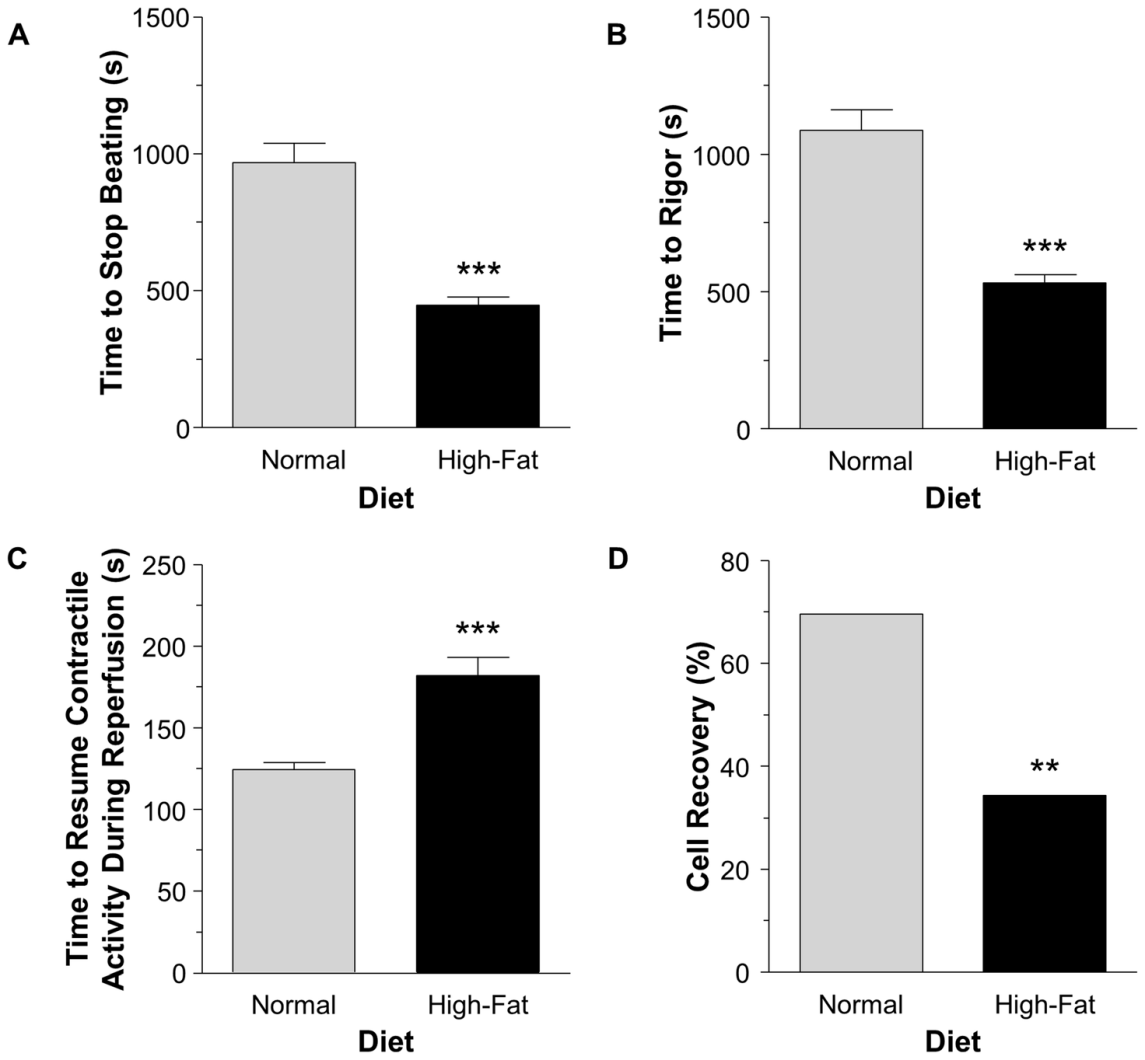
Cardiomyocyte observations and measurements during metabolic inhibition and reperfusion. Cardiomyocyte time to stop beating (A) and time to the start of rigor (B) following metabolic inhibition (2 mM NaCN in the absence of substrates). C) Time between the start of reperfusion and the resumption of contractile activity. D) Percentage of cardiomyocytes that had full recovery (beating without arrhythmia) following a period of metabolic inhibition. Data are presented as mean±SEM (n≥25 cardiomyocytes from at least 6 hearts). Data were analyzed using the Mann-Whitney U test (A–C) and Fisher's exact test (D). *** = P<0.001 and ** = P<0.01 vs. normal diet.

Superfused cardiomyocytes isolated from mice fed high-fat diet stopped beating and entered rigor during metabolic inhibition (2 mM NaCN, substrate-free buffer) at earlier times compared to cardiomyocytes from normal diet mice, P<0.001 (Figure 2 A-B). Upon reperfusion, after 10 min in rigor, cardiomyocytes regained beating (either beating or arrhythmic beating) quicker in the normal diet group compared to high-fat diet, P<0.001 (Figure 2 C) and regained full recovery (beating without arrhythmia) more often than the cardiomyocytes isolated from mice fed high-fat diet, P<0.01 (Figure 2 D).

### The effect of non-obesogenic high-fat diet on oxidative state and antioxidant proteins

Cardiac oxidative stress (MDA content) was significantly lower in hearts from mice fed high-fat diet compared to hearts from normal diet mice (5.02±0.13 vs. 5.70±0.14 nmol·mg wet tissue^−1^, respectively). There were only two antioxidant proteins which had altered expression level in the high-fat diet group compared to the normal diet group determined using proteomics: catalase (increase) and mitochondrial superoxide dismutase 2 (SOD-2) (decrease), P<0.05 (Table 3).

**Table 3:** Antioxidant proteins determined using proteomics.

Protein (Accession number)	Normal	High-Fat	Fold Change vs. Normal	P-Value
Catalase (A2AL20)	0.79±0.03	1.22±0.13	1.54	0.02*
Isoform cytoplasmic & peroxisomal of Peroxiredoxin 5 (P99029-2)	1.01±0.02	1.02±0.03	1.00	0.91
Peroxiredoxin 1 (P35700)	1.02±0.03	1.07±0.01	1.05	0.13
Peroxiredoxin 2 (Q61171)	0.99±0.02	1.03±0.04	1.03	0.51
Peroxiredoxin 6 (D3Z0Y2)	0.98±0.02	1.01±0.02	1.03	0.32
SOD-1 (P08228)	1.16±0.03	1.15±0.05	0.99	0.91
SOD-2 (P09671)	1.14±0.05	0.89±0.06	0.78	0.02*
Thioredoxin-dependent peroxide reductase, mitochondrial (P20108)	0.98±0.03	1.03±0.01	1.05	0.13

Data are presented as mean±SEM (n = 4 hearts) and are relative to the pooled sample and normalized to GAPDH. Data were analyzed using Student's t-test. * = P<0.05 vs. normal diet.

### The effect of non-obesogenic high-fat diet on ROS levels in isolated cardiomyocytes and mitochondria

ROS turnover, as measured using the rate of DCF oxidation, did not differ between the two groups when the cardiomyocytes were incubated in HEPES buffer containing either glucose or palmitate (Figure S3 A). Nor did hydrogen peroxide production (measured using Amplex red) by isolated mitochondria in state 3.5 oxidizing pyruvate plus L-malate or palmitoylcarnitine plus L-malate differ between the two groups (Figure S3 B).

### The effect of non-obesogenic high-fat diet on oxygen consumption by cardiac mitochondria in different respiratory states

The rate of oxygen consumption fuelled by NADH from pyruvate plus L-malate oxidation was similar for both groups in all tested respiration states (Figure S4 A). The oxygen consumption rates were also not different between the normal and high-fat diet groups when comparing the β-oxidation pathway using palmitoylcarnitine plus L-malate as substrates (Figure S4 A). The respiratory control ratio (state 3/state 2) was also not different between the normal and high-fat diet groups for both sets of substrates (Figure S4 B).

### The effect of non-obesogenic high-fat diet on mitochondrial morphology

**Figure 3:**
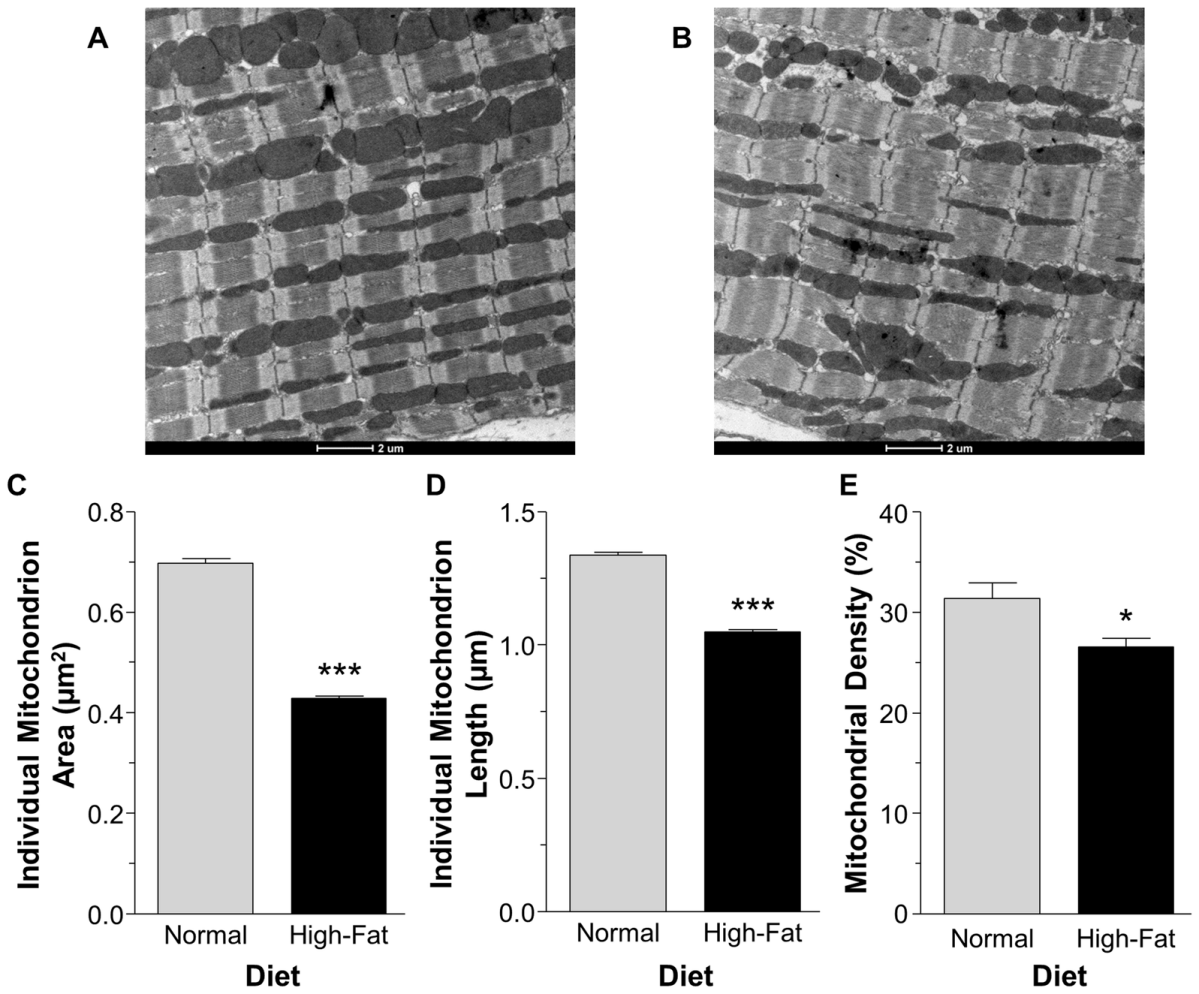
Representative electron micrographs of mitochondria and measurements. Representative electron micrographs from mice fed normal diet (A) and high-fat diet (B). Individual mitochondrion area (C) and length (D) and mitochondrial coverage of myofilament area (E) assessed using transmission electron micrographs. Data are presented as mean±SEM (n = 4 hearts and ≥900 mitochondria per heart from ≥10 electron micrographs per heart). Data were analyzed using Mann Whitney U test (C–D) and Student's t-test (E). *** = P<0.001 and * = P<0.05 vs. normal diet.

The mitochondria in the high-fat diet group were both smaller in area and shorter in length, P<0.001 (Figure 3). The total mitochondrial area, as a percentage of total myofilament area (referred to as mitochondrial density), was decreased in the high-fat diet group compared to the normal diet, P<0.05 (Figure 3).

### The effect of non-obesogenic high-fat diet on mitochondrial fusion and fission proteins

**Figure 4:**
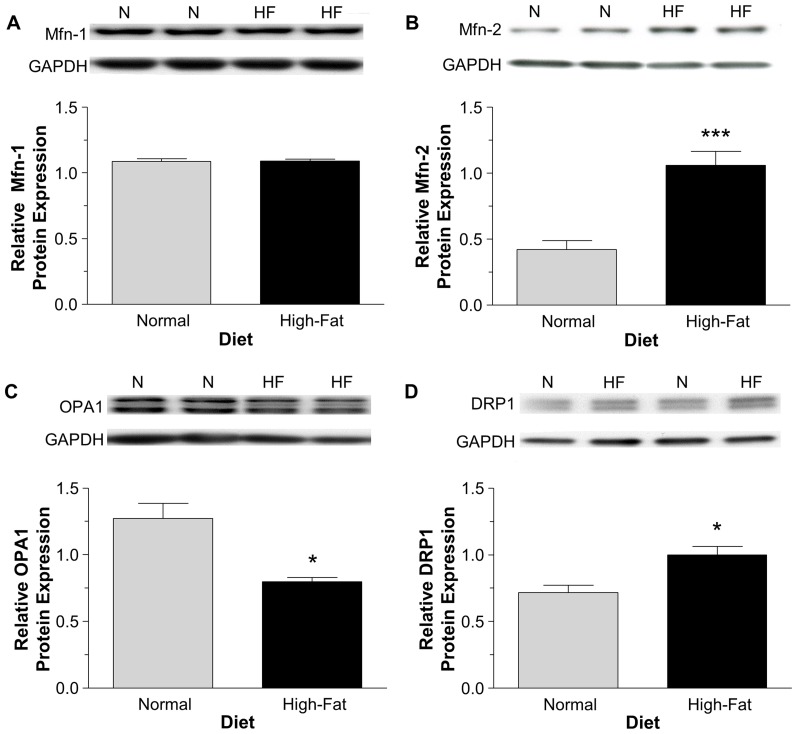
The relative cardiac protein expression of mitochondrial fusion and fission proteins determined using western blotting. The fusion proteins were Mfn-1 (A), Mfn-2 (B) and OPA1 (C) and the fission protein was DRP1 (D). Protein bands were normalized to GAPDH. Data are presented as mean±SEM (n = 6 hearts). Data were analyzed using Student's t-test (A, B and D) and Student's t-test with Welch's correction (C). *** = P<0.001 and * = P<0.05 vs. normal diet.

In response to the high-fat diet the expression of the fusion-related proteins Mfn-2 and OPA1 were significantly increased and decreased respectively, with no change in Mfn-1 expression but an increased expression of the fission-related protein, DRP1 (Figure 4).

### The effect of non-obesogenic high-fat diet on putative mPTP proteins

**Figure 5:**
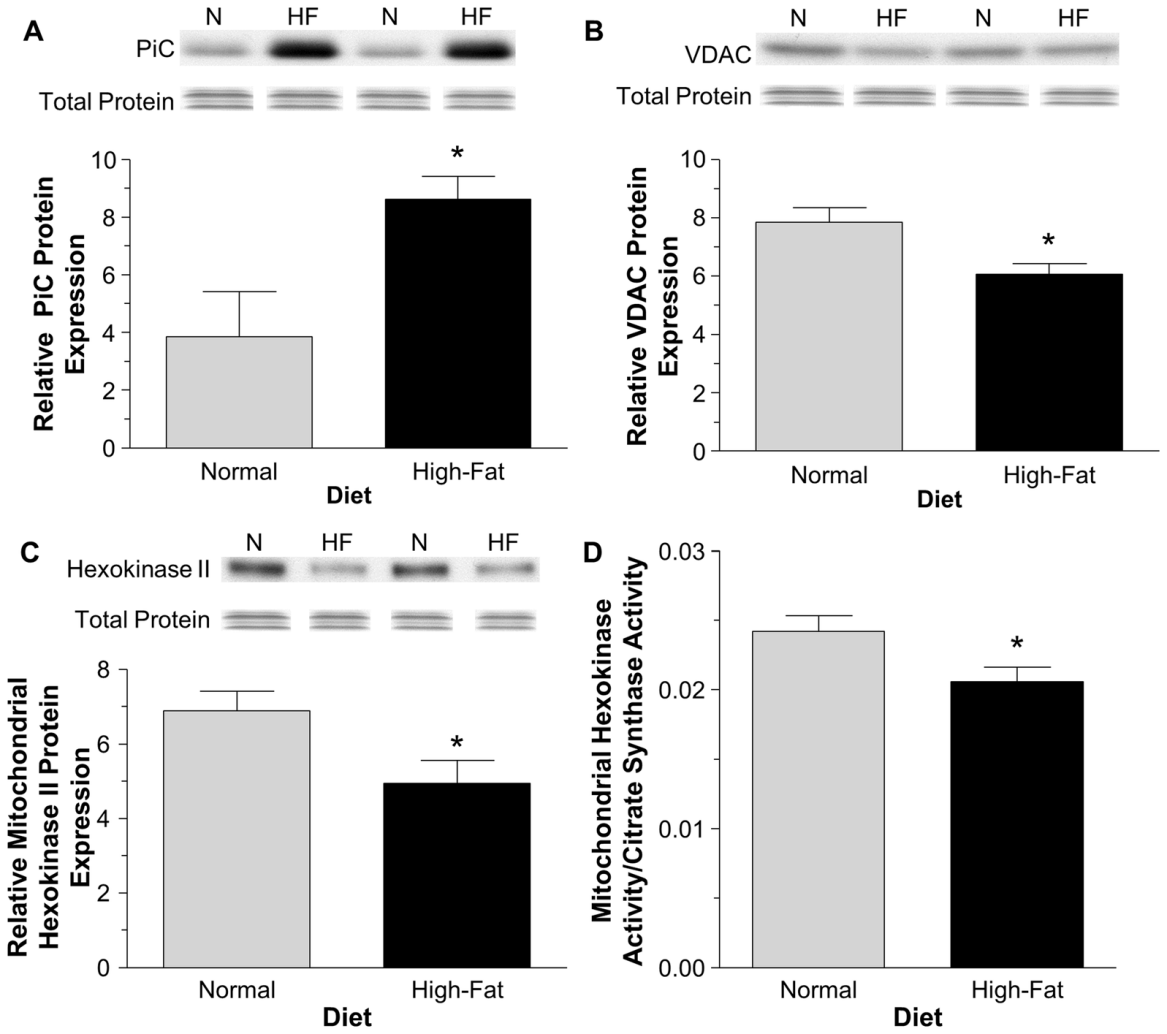
Relative cardiac protein expression of putative mitochondrial permeability transition pore components/regulators. PiC (A), VDAC (B), mitochondrial hexokinase II protein (C) and mitochondrial hexokinase activity (D). Data are presented as mean±SEM (n = 5 mitochondrial isolations). Data were analyzed using Student's t-test with Welch's correction (A) and Student's t-test (B–D). * = P<0.05 vs. normal diet.

Isolated cardiac mitochondria were assessed for the relative expression of putative mPTP proteins using western blotting. In the high-fat diet group there was a significant increase in the relative protein expression of PiC and a decrease in VDAC compared to the normal diet group, P<0.05 (Figure 5 A–B). There was no change in both CypD and ANT in the high-fat diet group compared to the normal diet group (Figure S5).

### The effect of non-obesogenic high-fat diet on mitochondrial hexokinase protein content and activity

There was less hexokinase II protein at the mitochondrial level in the high-fat diet group compared to the normal diet group, P<0.05 (Figure 5 C). The hexokinase activity at the mitochondrial level was also significantly lower in the high-fat diet group compared to the normal diet group, P<0.05 (Figure 5 D).

### The effect of non-obesogenic high-fat diet on diastolic intracellular Ca^2+^

**Figure 6:**
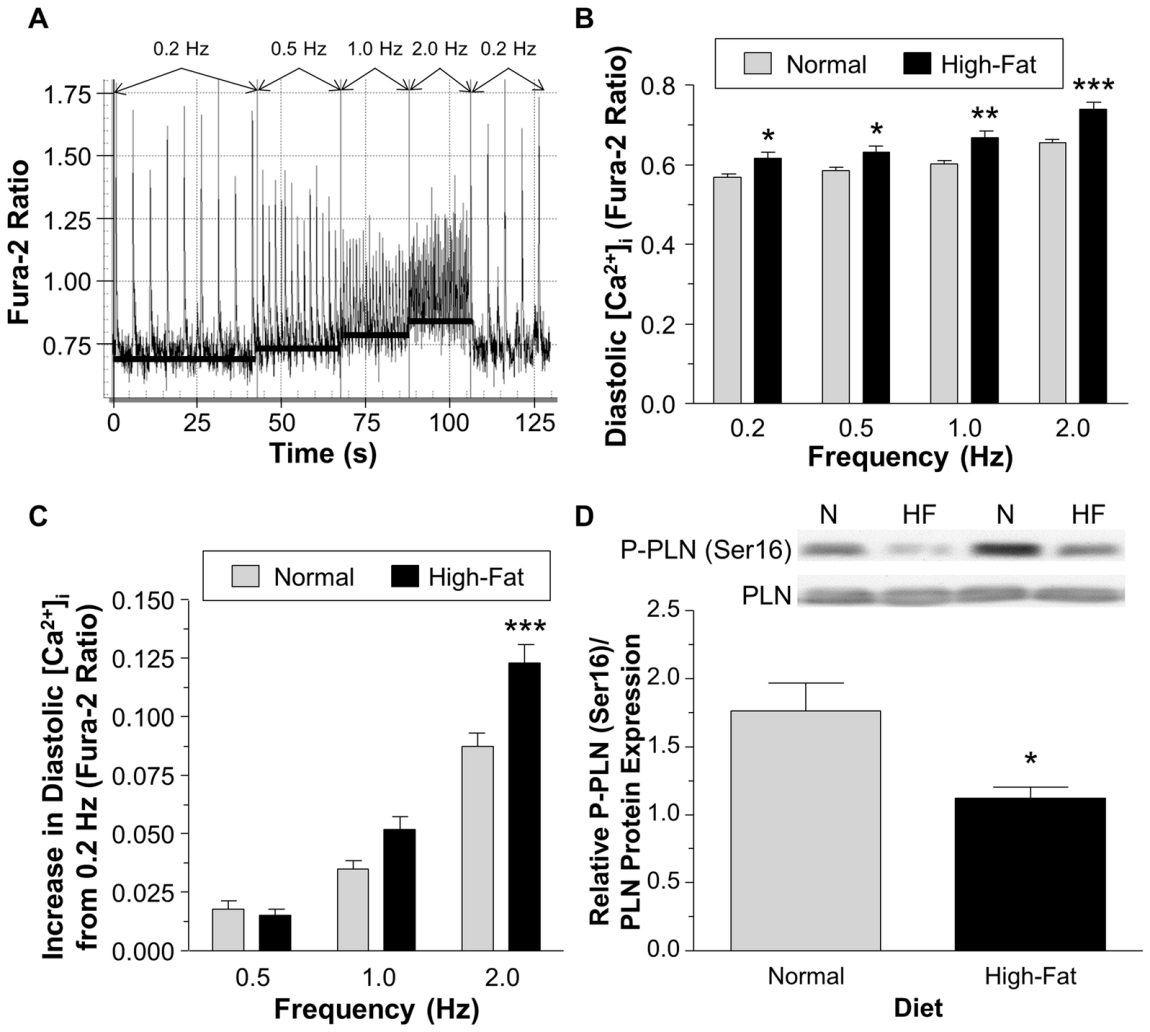
The diastolic intracellular Ca^2+^ concentration measured using Fura-2 AM fluorescence in isolated cardiomyocytes. A) An example trace of Ca^2+^ transients using the Fura-2 fluorescent dye. The cell was stimulated at the frequencies indicated. B) Diastolic [Ca^2+^]_i_ at different frequencies in isolated cardiomyocytes. C) Diastolic [Ca^2+^]_i_ with the 0.2 Hz diastolic [Ca^2+^]_i_ subtracted. D) The relative phosphorylated phospholamban (P-PLN Ser16) to phospholamban (PLN) protein expression ratio. Data are presented as mean±SEM (n = 29–32 cardiomyocytes from 4 hearts (B–C) and 6 hearts (D)). Data were analyzed using two-way ANOVA with the Bonferroni post-hoc test (B–C) and Student's t-test (D). *** = P<0.001, ** = P<0.01 and * = P<0.05 vs. normal diet.

The diastolic [Ca^2+^]_i_ in isolated cardiomyocytes was elevated in the high-fat diet group compared to the normal diet group at all frequencies tested, P<0.05 (Figure 6 B). To consider the difference at 0.2 Hz the diastolic [Ca^2+^]_i_ was also presented as diastolic [Ca^2+^]_i_ with the 0.2 Hz [Ca^2+^]_i_ subtracted. This showed that as the frequency increased the difference between the normal diet and high-fat diet became larger and became significant at 2.0 Hz (Figure 6 C). The level of phospholamban phosphorylation (Ser16) decreased in the high-fat diet compared to normal diet, P<0.05 (Figure 6 D).

### The changes in catalase, MDA and the efficacy of CsA during I/R

**Figure 7:**
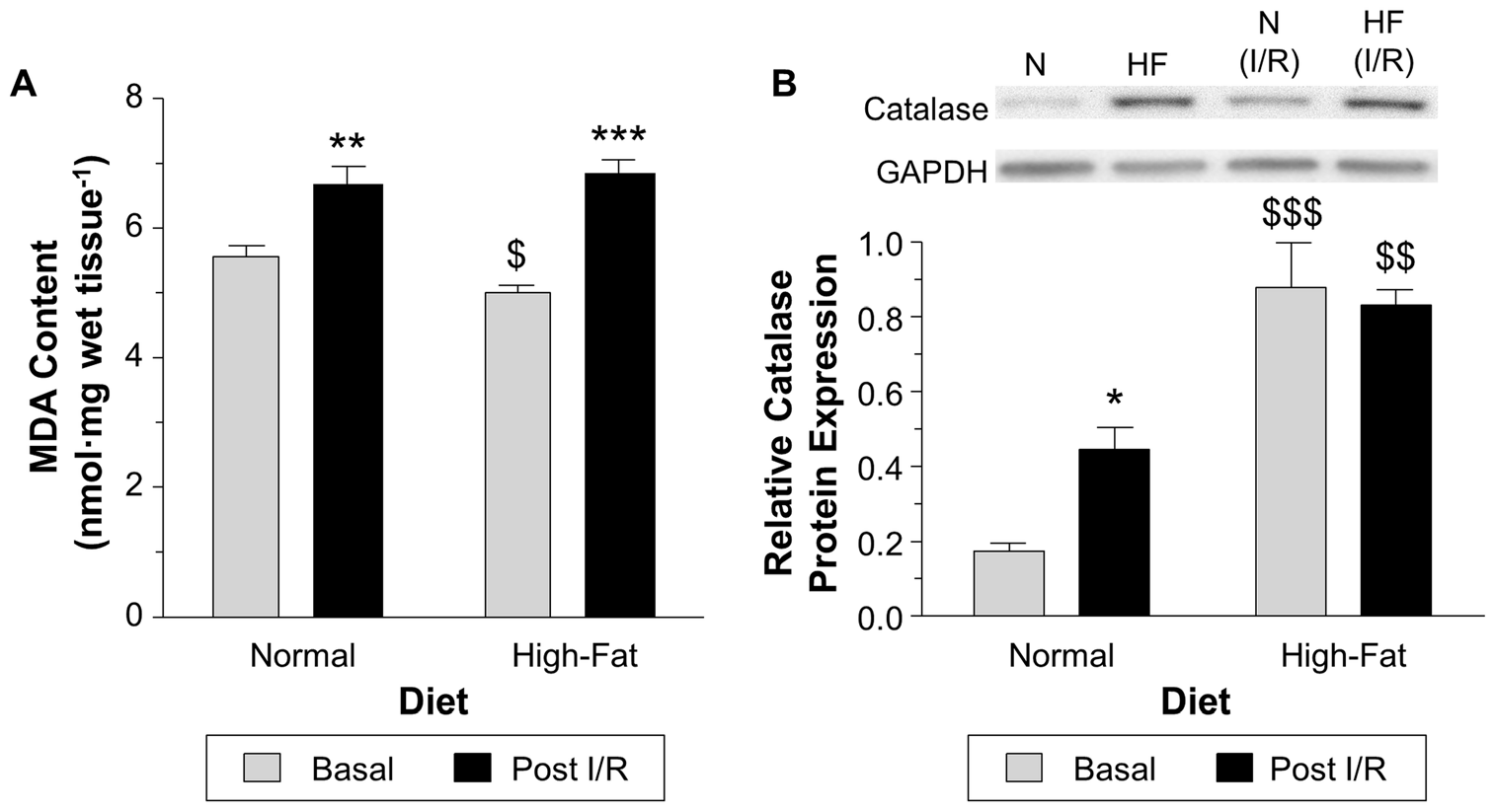
Oxidative state during I/R. A) MDA measurements in unperfused heart tissue or hearts collected at the end of an I/R protocol analyzed using HPLC. B) The relative catalase protein expression at basal level and after I/R determined using western blotting with all sample run on the same membrane. Data are presented as mean±SEM (n = 5–6 hearts (A) and 3 hearts (B)). Data were analyzed using two-way ANOVA with the Bonferroni post-hoc test (A). *** = P<0.001, ** = P<0.01, * = P<0.05 vs. basal, 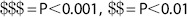 and $ = P<0.05 vs. normal diet.

Cardiac oxidative stress (MDA content) significantly increased at the end of I/R reaching similar levels for both hearts isolated from mice fed normal or high-fat diet (Figure 7 A). However, the relative change during I/R was more pronounced in the high-fat diet compared to the normal diet (increased by 18% and 36%, for normal and high-fat diet, respectively). In contrast, catalase expression which was significantly higher in hearts from high-fat diet did not change during I/R, whereas in the normal diet group there was a doubling of catalase at the end of I/R compared to basal levels, P<0.05 (Figure 7 B and S6).

**Figure 8:**
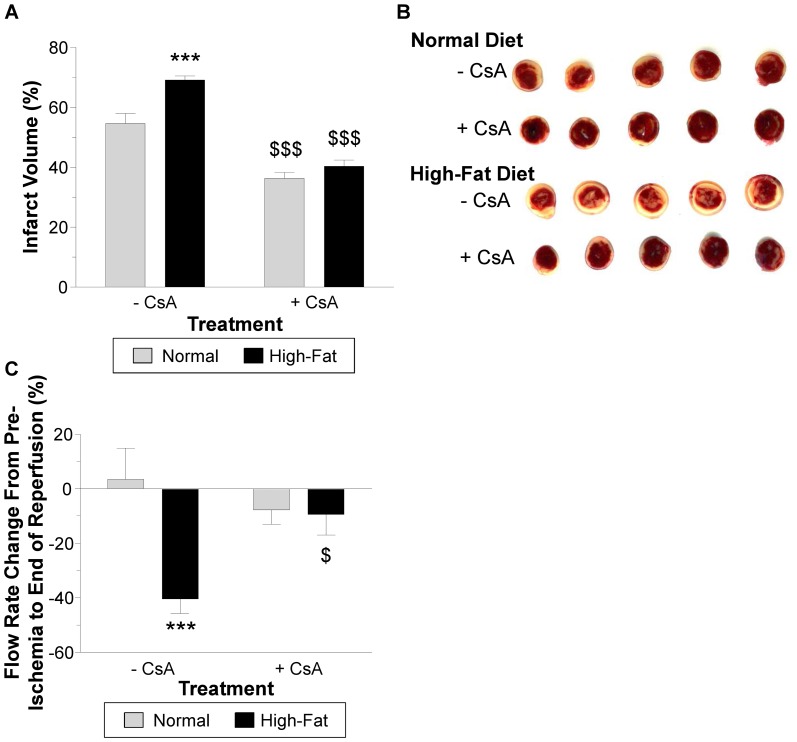
I/R injury in isolated hearts with and without the addition of 0.2 µM CsA. The CsA was added to the buffer 10) Infarct volume quantified from the heart slices stained with TTC. B) Representative heart slices from the normal diet and high-fat diet groups. C) The change in flow rate from pre-ischemia to the end of reperfusion. Data are presented as mean±SEM (n = 5-6 hearts). Data were analyzed using two-way ANOVA with the Bonferroni post-hoc test. *** = P<0.001, ** = P<0.01 vs. normal diet, $$$ = P<0.001 and $ = P<0.05 vs. CsA.

CsA significantly decreased infarct size in the normal diet and high-fat diet groups, P<0.001 (Figure 8 A–B). However the extent of the protection was more marked for high-fat diet (from 69.1±1.3 to 40.3±2.1% infarct volume) compared to normal diet (from 54.6±3.4 to 36.3±2.1% infarct volume). Finally, CsA did not alter the recovery in flow rate in the normal diet group whereas in the high-fat diet group the flow rate recovery after an I/R protocol was significantly improved (Figure 8 C).

## Discussion

The overwhelming majority of experimental studies investigating the effect of elevated plasma lipids on cardiac pump remodeling have used models of obesity where cellular, functional and structural cardiac changes can be attributed to obesity-associated co-morbidities. Subsequently, the reported increase in vulnerability to I/R of hearts from animals fed high-fat diet has been explained in terms of obesity related effects (e.g. diabetes, cardiac hypertrophy) as well as lipid-induced direct effects on the heart. What has been lacking thus far is a model of hyperlipidemia and hypercholesterolemia with no obesity to directly determine lipid-induced cardiac remodeling in the context of vulnerability to cardiac insults. In this study, we have shown for the first time that high-fat diet, independent of obesity-induced co-morbidities, increases the vulnerability of both isolated hearts and cardiomyocytes to cardiac insults. Furthermore, high-fat diet triggered cellular changes (raised [Ca^2+^]_i_, oxidative stress during I/R, hexokinase II dissociation from the mitochondria and mitochondrial fragmentation) that can contribute to increased vulnerability by altering mPTP opening during I/R.

### A non-obese mouse model of hyperlipidemia and hypercholesterolemia

In this study we characterized a mouse model of hyperlipidemia and hypercholesterolemia that is not obese and does not show key obesity-associated morbidities. The 20 week duration of high-fat feeding was not sufficient to induce diabetes, coronary disease, cardiac hypertrophy, apoptosis or alter cardiac pump function. In this model the body weight increased by 3% whereas models of obesity in mice see an increase in body weight to 40 g (30% increase) or more [52], [53]. This was achieved by feeding mice a high-fat, very low sucrose diet for 20 weeks. Using high sucrose instead of starch in normal or high-fat diet appears to be key in triggering significant weight gain/obesity ([33] and see Introduction). Consistent with this is the original work by Surwit *et al*. [54] showing significant obesity and diabetes in C57BL/6J mice using disaccharides as the primary source of carbohydrate.

### Increased vulnerability of hearts and cardiomyocytes to cardiac insults

Isolated hearts and cardiomyocytes from mice fed high-fat diet had increased vulnerability to cardiac insults compared to those fed normal diet. This is similar to earlier studies on animals fed high-fat diet causing obesity [18]. However, in this study the triggers will be largely due to hyperlipidemia induced cardiac cellular remodeling independent of obesity. The increased vulnerability to I/R in our study is unlikely to be due to the absence of fatty acids from the perfusate as an earlier study demonstrated increased vulnerability even in the presence of an *in vivo* circulating concentrations of lipids [25]. Myocardial I/R injury, which can occur during clinical procedures such as thrombolysis, angioplasty and coronary artery bypass graft surgery, is triggered by significant Ca^2+^ overload and oxidative stress that lead to mPTP opening [23], [24]. High-fat diet has been shown to induce oxidative stress [13] which may augment Ca^2+^ overload and I/R injury [55], [56].

### Non-obesogenic high-fat diet increases vulnerability to oxidative stress during I/R

Cardiac oxidative stress and increased catalase levels are closely linked to increased fat metabolism associated with high-fat diet and obesity [57]–[59]. A surprising finding in this study was that feeding mice non-obesogenic high-fat diet for 20 weeks actually lowered the level of myocardial lipid peroxidation compared to control. Decreased ROS in other tissues from high-fat feeding has already been reported and has been attributed to increasing proliferation of peroxisomes and thus catalase levels [60]. The marked increase in cardiac catalase expression in our high-fat diet model (nearly 5 fold, Figure 7) is likely to be responsible for the relatively lower MDA levels. Apart from a fall in SOD-2 there was no difference in the expression of other antioxidant enzymes (Table 3).

Both lower cardiac MDA and higher catalase are expected to render the heart more resistant to I/R injury. However, we found hearts from high-fat diet mice were more vulnerable to I/R and less able to cope with oxidative stress as shown by inability to increase the levels of catalase compared to normal diet (Figure 7). Thus it would appear that hearts of mice fed non-obesogenic high-fat diet had already upregulated catalase in response to the diet and therefore were not able to adapt to a further increase in oxidative stress during I/R. In contrast, and because the basal level of cardiac catalase was relatively low, hearts from normal diet mice were able to increase catalase protein expression during I/R. This work clearly suggests that elevated catalase prior to I/R is only important if the levels of the enzyme can be further augmented during the cardiac insult.

### Non-obesogenic high-fat diet triggers mitochondrial fragmentation and disruption to Ca^2+^ cycling

Myocardial mitochondria are a major source for ROS production during I/R (reviewed in [61]) and any changes in their structure and function during high-fat feeding might impact on ROS production and vulnerability to I/R. Our data indicate that despite changes to substrate supply in high-fat diet group, isolated mitochondria retain the capacity to oxidize different substrates to a similar level as control (Figure S4). These findings are similar to those reported for high-fat diet induced obesity models [62], [63], although there are reports indicating differences in oxygen consumption [64].

The interfibrillar mitochondria from mice fed a high-fat diet were smaller, shorter and covered less myofilament area compared to the normal diet group which is suggestive of mitochondrial fission. However, the changes observed in fusion and fission proteins did not present a clear picture of how this might be achieved. Reduced OPA1 indicates fission although there are reports suggesting less OPA1 increases the size of the mitochondria [65]. Elevated Mfn-2 levels are also indicative of fission as Mfn-2 knockout mouse have been reported have larger and longer mitochondria [66]. Mfn-2 is involved in tethering the endoplasmic reticulum with the mitochondria [67] and this might create a pool of higher concentration of Ca^2+^ around the mitochondria. Elevated Ca^2+^ has been reported to recruit DRP1 to mitochondria and induce fission [68] as well as making them more likely to experience Ca^2+^ overload [69]. Consistent with this, cardiomyocytes from the Mfn-2 knockout mice are more resistant to simulated I/R [70]. It has been proposed that larger mitochondria are able to accommodate Ca^2+^ loading better during I/R and therefore reduce cell death [71] whilst mitochondrial fragmentation increases rate of ROS production [68], [72]. Overall, the increase in cardiac Mfn-2 in high-fat diet is consistent with increased fission and vulnerability to I/R.

In addition to ROS generation, Ca^2+^ overload is also a key determinant of I/R injury. Cardiomyocytes from high-fat diet had higher diastolic [Ca^2+^]_i_ compared to normal diet (Figure 6). The finding that these hearts have reduced phosphorylated phospholamban suggests that it may be impairment in sarcoplasmic reticulum function that leads to higher levels of [Ca^2+^]_i_. Generation of ROS has been implicated in Ca^2+^ handling defects including depressed Ca^2+^ uptake by the sarcoplasmic reticulum [73], [74]. The impairment of coronary flow in the isolated hearts during reperfusion in the high-fat diet group is likely to be a result of increase in vascular resistance caused by increased contracture (diastolic dysfunction). Diastolic dysfunction during reperfusion is strongly linked to Ca^2+^ overload [75] which is likely to lead to a raised mitochondrial Ca^2+^ content. Along with ROS production, Ca^2+^ loading would render the mPTP more prone to opening during I/R [76].

### A role for the mitochondrial permeability transition pore in increased vulnerability to I/R

Cardiac remodeling in response to high-fat diet indicates changes that would increase mPTP opening. However, there was also direct evidence showing significant changes in the expression levels of mitochondrial proteins implicated as components and regulators of the mPTP. High-fat diet reduced mitochondrial hexokinase II activity and expression compared to the normal diet group. Hexokinase II is a metabolic sensor and therefore altering the diet would change the substrate supply which could alter the localization of hexokinase II [77]. Mitochondrial hexokinase II content is important during I/R injury as the amount at the end of ischemia negatively correlates with infarct size [50]. The loss of mitochondrial hexokinase II is thought to destabilize mitochondrial contact sites which induces outer mitochondrial membrane permeability [50].

Key putative components of mPTP were also altered by high-fat diet. There was a significant decrease in the expression of VDAC, an increase in mitochondrial PiC but no change in CypD and ANT. Not only does the PiC transport phosphate into the matrix of the mitochondria where it can activate the mPTP; it has also been proposed to be a component of the mPTP structure [76], [78]. VDAC is the proposed binding site for hexokinase [79]. Therefore a reduction in VDAC protein might be a reason for the decreased amount of hexokinase II binding at the mitochondria. Whether changes to the components of the mPTP would have an impact on the amount of mPTP opening is not presently known. However, opening of the mPTP can lead to further ROS production through loss of cytochrome c and this can generate a positive feedback loop of ROS formation and mPTP opening [51].

Our data are consistent with the proposal that feeding mice non-obesogenic high-fat diet for a period of 20 weeks triggers cardiac remodeling which increases the sensitivity of the mPTP to open during I/R. This is consistent with CsA showing relatively more efficacy in protecting the heart in high-fat diet group compared to the normal diet group.

In conclusion, feeding mice non-obesogenic high-fat diet increases the vulnerability of both isolated hearts and cardiomyocytes to cardiac insults. A number of factors (raised [Ca^2+^]_i_, oxidative stress during I/R, hexokinase II dissociation from the mitochondria and mitochondrial fragmentation) in the high-fat diet group can contribute to this effect possibly by altering mPTP opening during I/R [50], [76], [80].

## Supporting Information

Figure S1 **Representative histology and echocardiography images.** Representative aortic sinus (A), brachiocephalic artery (B) and coronary arteries (C) from mice fed high-fat diet. Sections are stained with elastic van Gieson; purple = elastin and red = connective tissue. Arrow head points to elastin. Scale bars = 200 µm. Representative echocardiographic traces from normal diet (D) and high-fat diet (E) mice. M-mode echocardiographic measurements were taken in the parasternal short axis view at the level of the papillary muscles.
(TIF)

Figure S2 **The relative cardiac protein expression of apoptotic markers determined using western blotting.** The relative protein expression of P-Akt (Ser473)/Akt ratio (A), cleaved-caspase 3 (CC3) (B), mitochondrial BAX (C) and BAX/Bcl-2 ratio (D) normalized to GAPDH or total protein blots. Data are presented as mean±SEM (n = 5–6 hearts). Data were analyzed using the Mann-Whitney U test (A–B and D) and Student's t-test (C). There was no statistical significance between the data.
(TIF)

Figure S3 **Reactive oxygen species production in isolated cardiomyocytes and isolated mitochondria.** A) DCF fluorescence measurements in quiescent cardiomyocytes using HEPES buffer containing either glucose or palmitate. B) Hydrogen peroxide production in isolated cardiac mitochondria using either pyruvate/L-malate (P/M) or palmitoylcarnitine/L-malate (Pal-Car). Data are presented as mean±SEM (n = 5-7 isolations). Data were analyzed using the Mann-Whitney U test (A) and Student's t-test (B). There was no statistical significance between the data.
(TIF)

Figure S4 **Mitochondrial oxygen consumption in different respiration states using different energy substrates.** Oxygen consumption was measured using an Oxygraph. A) Oxygen consumption in state 2, state 3.5 and state 3. The respiratory control ratio was calculated using the state 3:state 2 ratio (B). P/M = pyruvate/L-malate and Pal-Car = palmitoylcarnitine/L-malate. Data are presented as mean±SEM (n = 5 mitochondrial isolations). Data were analyzed using Student's t-test. There was no statistical significance between the data.
(TIF)

Figure S5 **Relative cardiac protein expression of putative mitochondrial permeability transition pore components/regulators determined using western blotting.** CypD (A) and ANT (B) normalized to total protein blots. Data are presented as mean±SEM (n = 5 mitochondrial isolations). Data were analyzed using Student's t-test (A) and the Mann-Whitney U test (B). There was no statistical significance between the data.
(TIF)

Figure S6 **The relative cardiac catalase protein levels at the basal level and after I/R.** Basal levels of catalase (A) and catalase after I/R (B) normalized to GAPDH. Data are presented as mean±SEM (n = 6 hearts). Data were analyzed using Student's t-test with Welch's correction. *** = P<0.001 and * = P<0.05 vs. normal diet.
(TIF)

Table S1 **High-fat diet (Special Diets Services code: 821424) formulation and specification data for guidance.**
(DOCX)
