# Supplementary figures and images for: Hearts from Mice Fed a Non-Obesogenic High-Fat Diet Exhibit Changes in Their Oxidative State, Calcium and Mitochondria in Parallel with Increased Susceptibility to Reperfusion Injury
Source: PLoS One. 2014 Jun 20;9(6):e100579. doi: 10.1371/journal.pone.0100579 (PMC4065057; doi:10.1371/journal.pone.0100579)

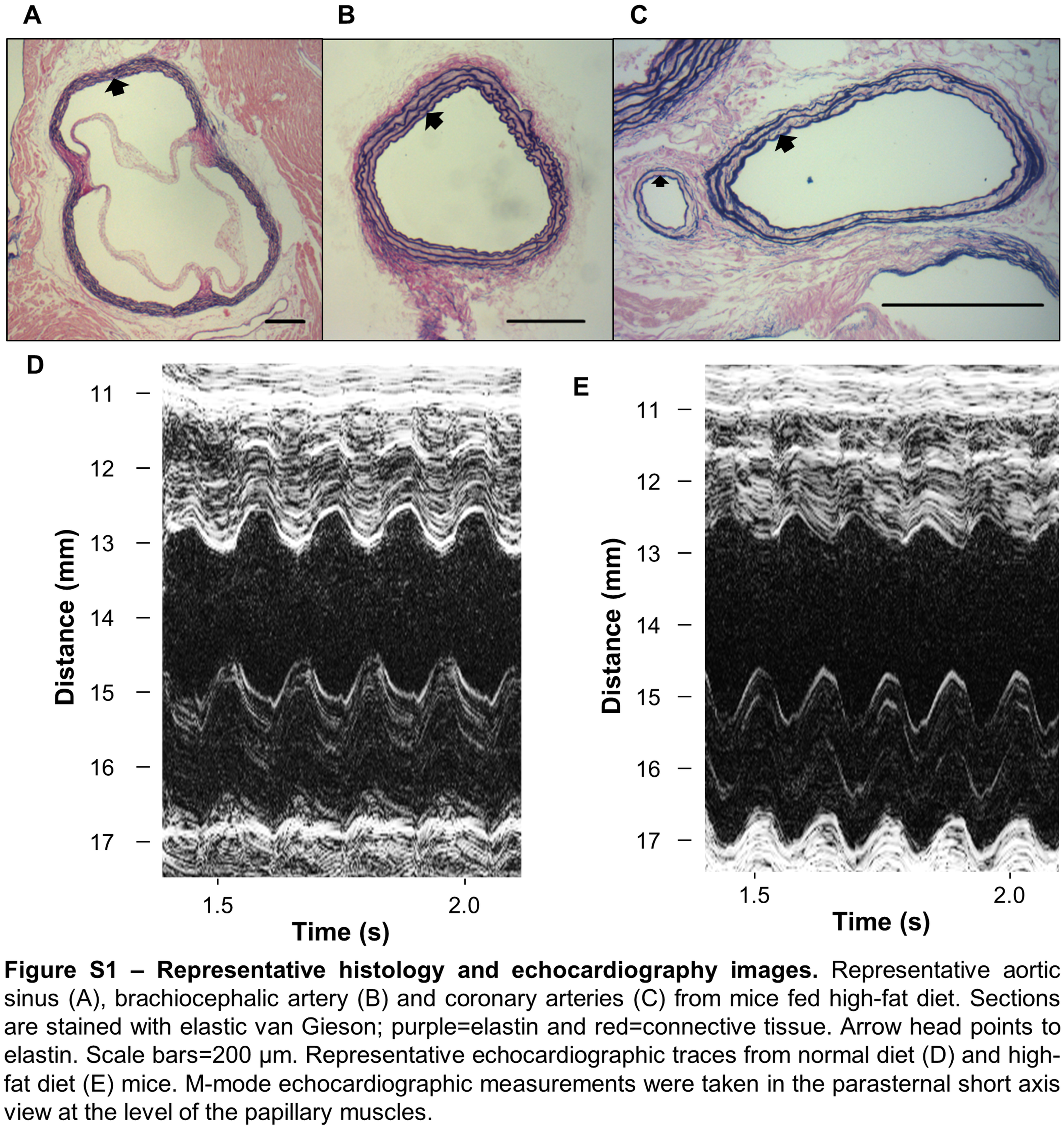

Supplement: Figure S1 — Representative histology and echocardiography images. Representative aortic sinus (A), brachiocephalic artery (B) and coronary arteries (C) from mice fed high-fat diet. Sections are stained with elastic van Gieson; purple = elastin and red = connective tissue. Arrow head points to elastin. Scale bars = 200 µm. Representative echocardiographic traces from normal diet (D) and high-fat diet (E) mice. M-mode echocardiographic measurements were taken in the parasternal short axis view at the level of the papillary muscles. (TIF) [file pone.0100579.s001.tif]

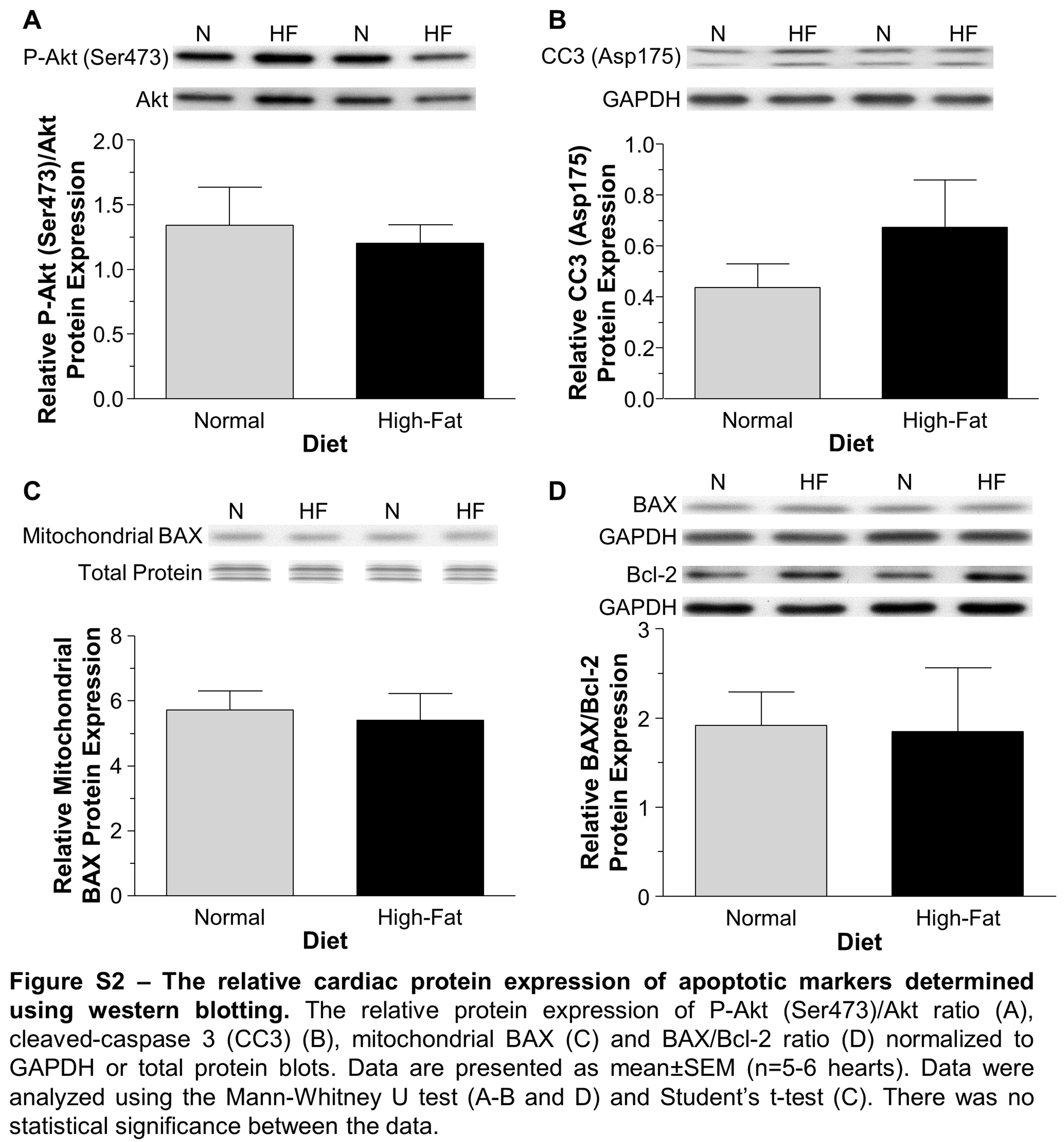

Supplement: Figure S2 — The relative cardiac protein expression of apoptotic markers determined using western blotting. The relative protein expression of P-Akt (Ser473)/Akt ratio (A), cleaved-caspase 3 (CC3) (B), mitochondrial BAX (C) and BAX/Bcl-2 ratio (D) normalized to GAPDH or total protein blots. Data are presented as mean±SEM (n = 5–6 hearts). Data were analyzed using the Mann-Whitney U test (A–B and D) and Student's t-test (C). There was no statistical significance between the data. (TIF) [file pone.0100579.s002.tif]

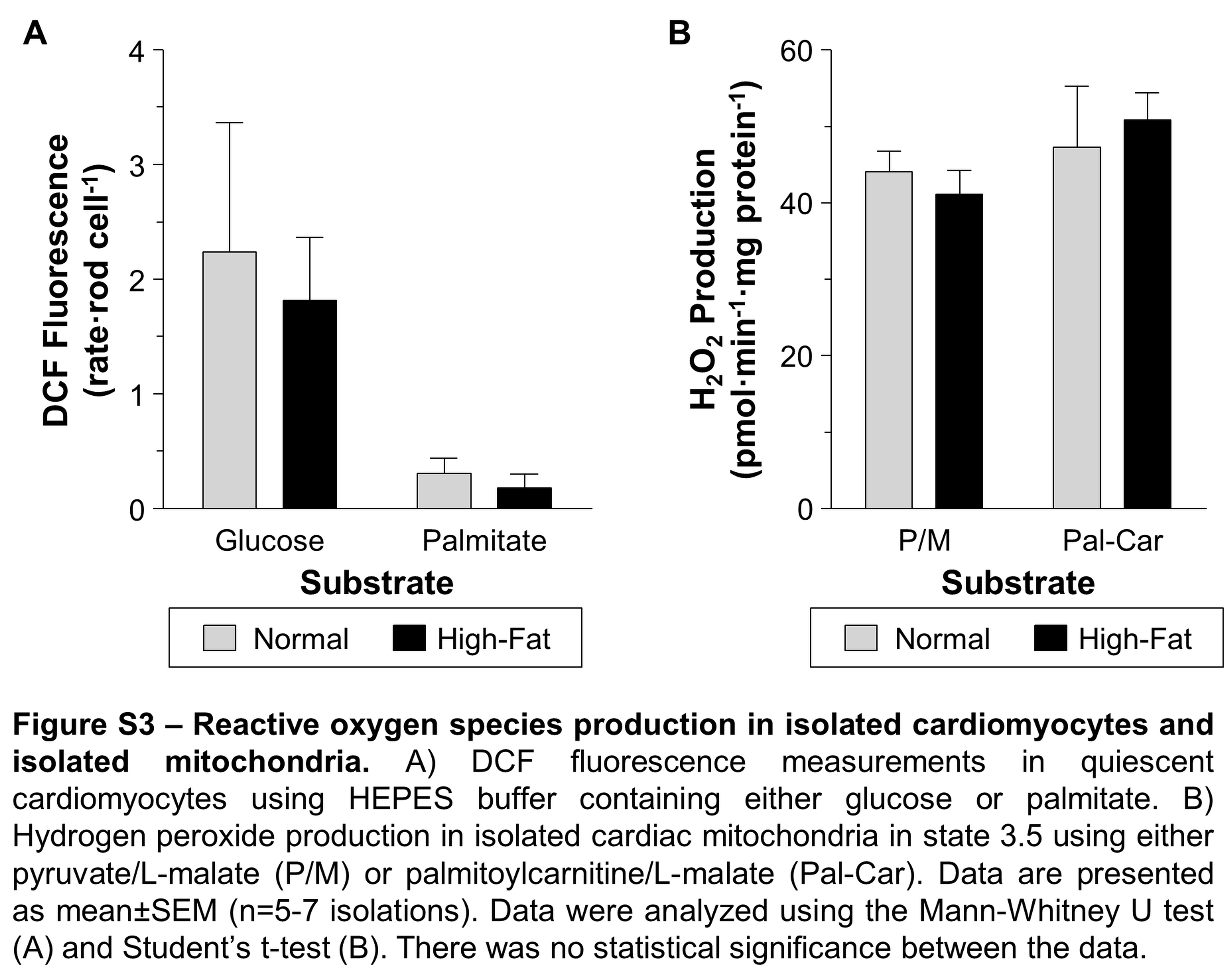

Supplement: Figure S3 — Reactive oxygen species production in isolated cardiomyocytes and isolated mitochondria. A) DCF fluorescence measurements in quiescent cardiomyocytes using HEPES buffer containing either glucose or palmitate. B) Hydrogen peroxide production in isolated cardiac mitochondria using either pyruvate/L-malate (P/M) or palmitoylcarnitine/L-malate (Pal-Car). Data are presented as mean±SEM (n = 5-7 isolations). Data were analyzed using the Mann-Whitney U test (A) and Student's t-test (B). There was no statistical significance between the data. (TIF) [file pone.0100579.s003.tif]

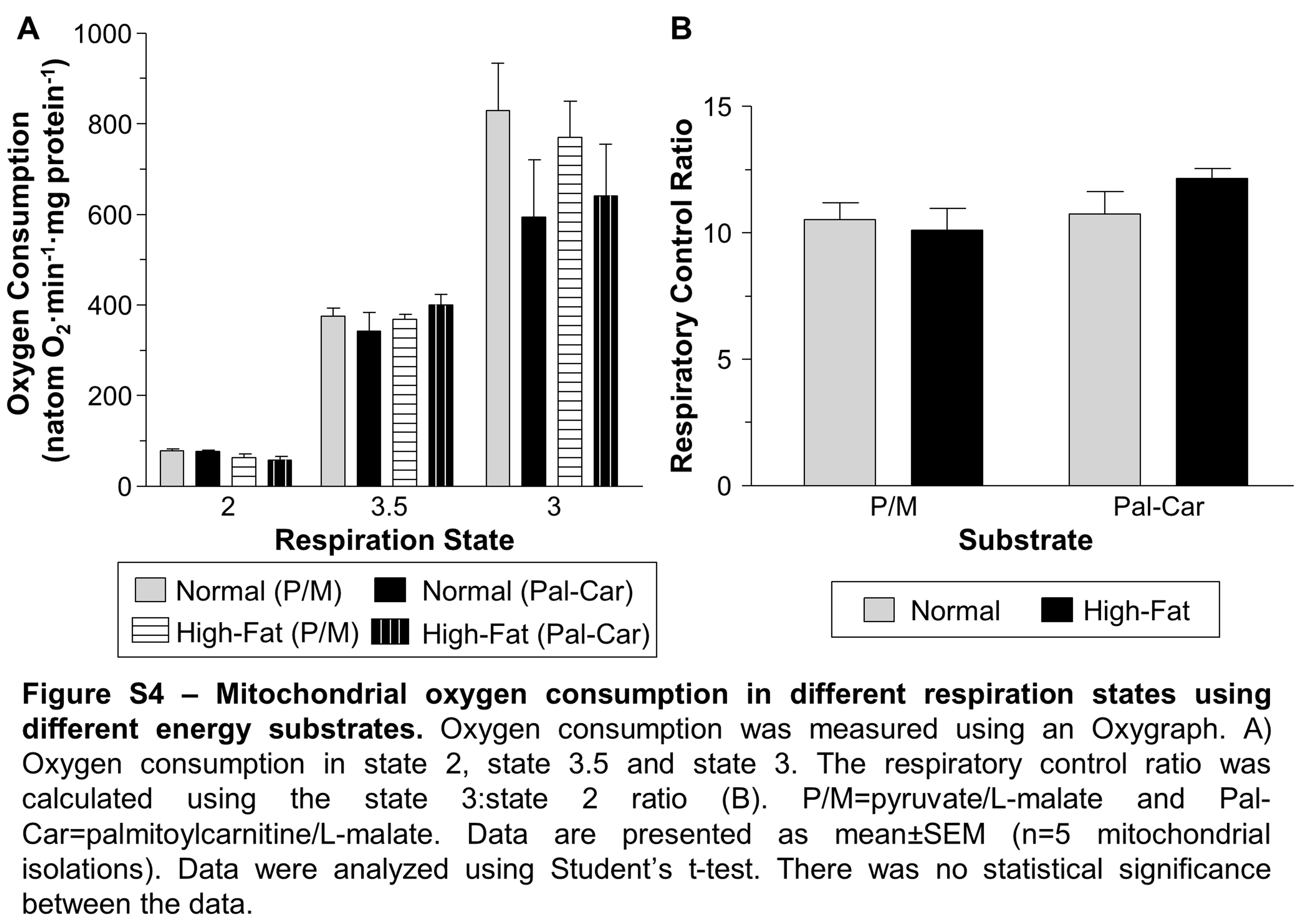

Supplement: Figure S4 — Mitochondrial oxygen consumption in different respiration states using different energy substrates. Oxygen consumption was measured using an Oxygraph. A) Oxygen consumption in state 2, state 3.5 and state 3. The respiratory control ratio was calculated using the state 3:state 2 ratio (B). P/M = pyruvate/L-malate and Pal-Car = palmitoylcarnitine/L-malate. Data are presented as mean±SEM (n = 5 mitochondrial isolations). Data were analyzed using Student's t-test. There was no statistical significance between the data. (TIF) [file pone.0100579.s004.tif]

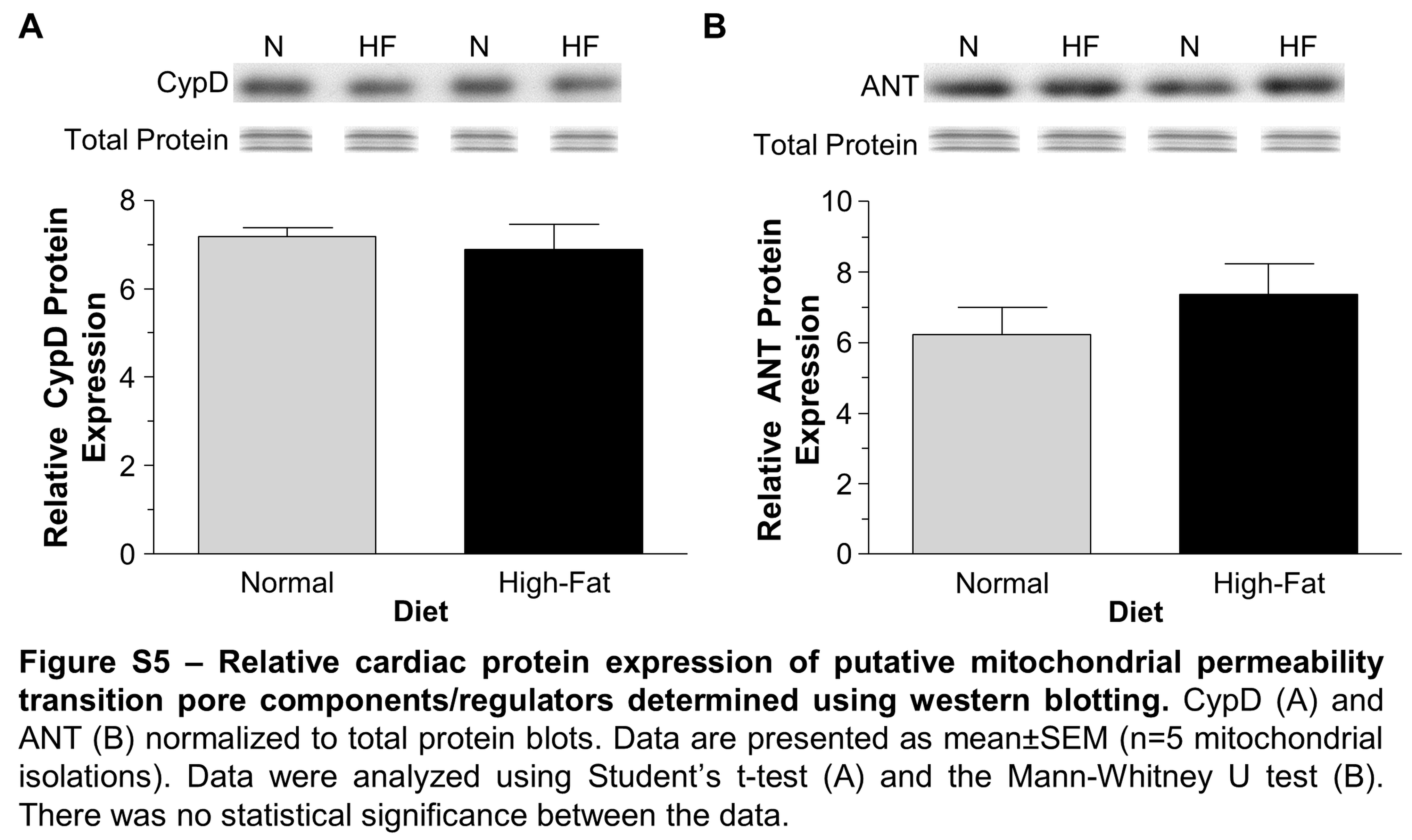

Supplement: Figure S5 — Relative cardiac protein expression of putative mitochondrial permeability transition pore components/regulators determined using western blotting. CypD (A) and ANT (B) normalized to total protein blots. Data are presented as mean±SEM (n = 5 mitochondrial isolations). Data were analyzed using Student's t-test (A) and the Mann-Whitney U test (B). There was no statistical significance between the data. (TIF) [file pone.0100579.s005.tif]

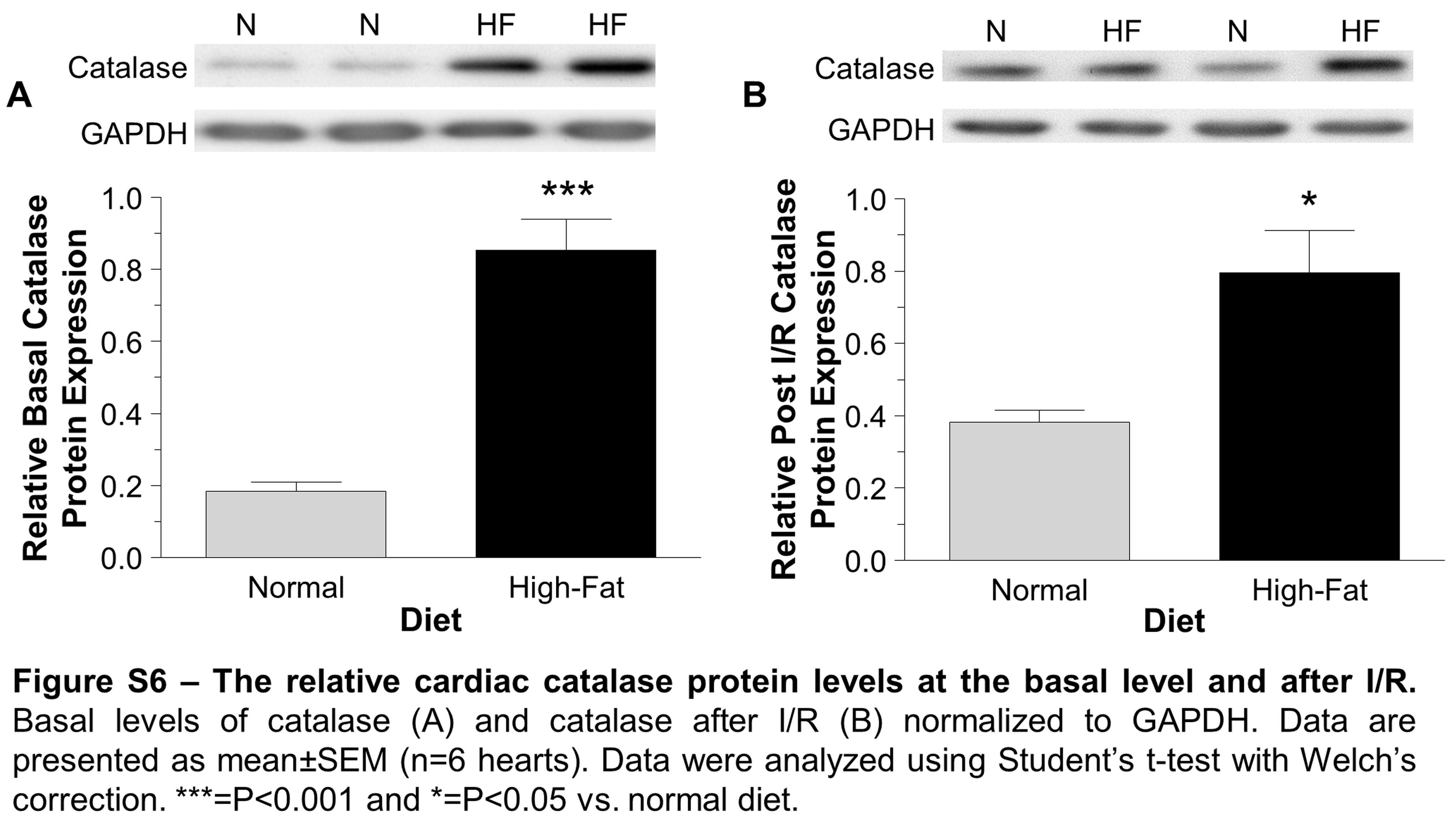

Supplement: Figure S6 — The relative cardiac catalase protein levels at the basal level and after I/R. Basal levels of catalase (A) and catalase after I/R (B) normalized to GAPDH. Data are presented as mean±SEM (n = 6 hearts). Data were analyzed using Student's t-test with Welch's correction. *** = P<0.001 and * = P<0.05 vs. normal diet. (TIF) [file pone.0100579.s006.tif]
