# Supplementary material for: Hearts from Mice Fed a Non-Obesogenic High-Fat Diet Exhibit Changes in Their Oxidative State, Calcium and Mitochondria in Parallel with Increased Susceptibility to Reperfusion Injury
Source: PLoS One. 2014 Jun 20;9(6):e100579. doi: 10.1371/journal.pone.0100579 (PMC4065057; doi:10.1371/journal.pone.0100579)
Supplement: Table S1 — High-fat diet (Special Diets Services code: 821424) formulation and specification data for guidance. (DOCX) [file pone.0100579.s007.docx]

**Table S1:** High Fat Diet (Special Diets Services code: 821424) formulation and specification data for guidance.

| **CALCULATED ANALYSIS:** | | |  |  |  |  |  |  |
| --- | --- | --- | --- | --- | --- | --- | --- | --- |
|  | Units | Fresh | | 10% H2O |  | Units | Fresh | 10% H2O |
| TOTAL | % | 100.00 | | 100.00 | TAURINE | % | 0.00 | 0.00 |
| MOISTURE | % | 7.46 | | 10.00 | GLYCINE | % | 0.83 | 0.81 |
| CRUDE OIL | % | 22.27 | | 21.66 | ASPARTIC ACID | % | 1.09 | 1.06 |
| CRUDE PROTEIN | % | 19.87 | | 19.32 | GLUTAMIC ACID | % | 3.34 | 3.25 |
| CRUDE FIBRE | % | 3.91 | | 3.80 | PROLINE | % | 1.40 | 1.36 |
| ASH | % | 5.24 | | 5.10 | SERINE | % | 0.79 | 0.77 |
| NFE | % | 41.02 | | 39.89 | HYD. PROLINE | % | 0.01 | 0.01 |
| PECTIN | % | 0.46 | | 0.45 | HYD. LYSINE | % | 0.00 | 0.00 |
| HEMICELLULOSE | % | 3.17 | | 3.08 | ALANINE | % | 0.57 | 0.55 |
| CELLULOSE | % | 4.85 | | 4.72 | Ca | % | 0.66 | 0.64 |
| LIGNIN | % | 0.43 | | 0.42 | P TOTAL | % | 0.57 | 0.55 |
| STARCH | % | 34.74 | | 33.79 | P PHYTATE | % | 0.05 | 0.05 |
| SUGAR | % | 1.24 | | 1.21 | P AVAILABLE | % | 0.52 | 0.51 |
| SDS GROSS ENERGY | MJ/kg | 19.67 | | 19.13 | Na | % | 0.23 | 0.22 |
| SDS DIGESTIBLE ENERGY | MJ/kg | 17.66 | | 17.18 | Cl | % | 0.38 | 0.37 |
| SDS METABOLISABLE ENERGY | MJ/kg | 16.54 | | 16.09 | K | % | 0.65 | 0.63 |
| AFE ENERGY | MJ/kg | 18.56 | | 18.05 | Mg | % | 0.17 | 0.17 |
| C14 1 MYRISTOLEIC | % | 0.03 | | 0.03 | Fe | mg/kg | 107.84 | 104.88 |
| C16 1 PALMITOLEIC | % | 0.11 | | 0.11 | Cu | mg/kg | 15.32 | 14.90 |
| C18 1 W9 OLEIC | % | 6.69 | | 6.51 | Mn | mg/kg | 61.83 | 60.13 |
| C18 2 W6 LINOLEIC | % | 1.95 | | 1.90 | Zn | mg/kg | 53.72 | 52.25 |
| C18 3 W3 LINOLENIC | % | 0.11 | | 0.11 | Co | μg/kg | 537.57 | 522.81 |
| C20 4 W6 ARICHIDONIC | % | 0.03 | | 0.03 | I | μg/kg | 12.74 | 12.39 |
| C22 5 W3 CLUPANODONIC | % | 0.00 | | 0.00 | Se | μg/kg | 45.28 | 44.04 |
| C12:0 LAURIC | % | 0.03 | | 0.03 | F | mg/kg | 4.67 | 4.54 |
| C14:0 MYRISTIC | % | 0.37 | | 0.36 | VITAMIN A | iu/kg | 6575.48 | 6395.00 |
| C16:0 PALMITIC | % | 4.56 | | 4.43 | VITAMIN D3 | iu/kg | 8050.00 | 7829.05 |
| C18:0 STEARIC | % | 2.04 | | 1.98 | VITAMIN E | iu/kg | 56.95 | 55.39 |
| ARGININE | % | 0.71 | | 0.69 | VITAMIN B1 THIAMINE | mg/kg | 6.52 | 6.34 |
| LYSINE | % | 1.16 | | 1.13 | VITAMIN B2 RIBOFLAVIN | mg/kg | 4.64 | 4.51 |
| METHIONINE | % | 0.48 | | 0.47 | VITAMIN B6 PYRIDOXIN | mg/kg | 5.82 | 5.66 |
| CYSTINE | % | 0.09 | | 0.09 | VITAMIN B12 CYANOCOBALAMINE | μg/kg | 5.45 | 5.30 |
| TRYPTOPHAN | % | 0.18 | | 0.18 | VITAMIN C ASCORBIC ACID | mg/kg | 7.97 | 7.75 |
| HISTIDINE | % | 0.48 | | 0.47 | VITAMIN K | mg/kg | 4.91 | 4.78 |
| THREONINE | % | 0.70 | | 0.68 | FOLIC ACID | mg/kg | 0.15 | 0.15 |
| ISOLEUCINE | % | 0.97 | | 0.94 | NICOTINIC ACID | mg/kg | 21.44 | 20.85 |
| LEUCINE | % | 1.60 | | 1.56 | PANTOTHENIC ACID | mg/kg | 13.30 | 12.93 |
| PHENYLALANINE | % | 0.85 | | 0.83 | CHOLINE | mg/kg | 278.07 | 270.44 |
| VALINE | % | 1.17 | | 1.14 | INOSITOL | mg/kg | 908.50 | 883.56 |
| TYROSINE | % | 0.83 | | 0.81 | BIOTIN | μg/kg | 56.35 | 54.80 |
